# *In silico* Experimentation of Glioma Microenvironment Development and Anti-tumor Therapy

**DOI:** 10.1371/journal.pcbi.1002355

**Published:** 2012-02-02

**Authors:** Yu Wu, Yao Lu, Weiqiang Chen, Jianping Fu, Rong Fan

**Affiliations:** 1 Department of Biomedical Engineering, Yale University, New Haven, Connecticut, United States of America; 2 Department of Mechanical Engineering, University of Michigan, Ann Arbor, Michigan, United States of America; 3 Human and Translational Immunology Program, Yale University, New Haven, Connecticut, United States of America; 4 Yale Comprehensive Cancer Center, Yale University, New Haven, Connecticut, United States of America; National University of Singapore, Singapore

## Abstract

Tumor cells do not develop in isolation, but co-evolve with stromal cells and tumor-associated immune cells in a tumor microenvironment mediated by an array of soluble factors, forming a complex intercellular signaling network. Herein, we report an unbiased, generic model to integrate prior biochemical data and the constructed brain tumor microenvironment *in silico* as characterized by an intercellular signaling network comprising 5 types of cells, 15 cytokines, and 69 signaling pathways. The results show that glioma develops through three distinct phases: pre-tumor, rapid expansion, and saturation. We designed a microglia depletion therapy and observed significant benefit for virtual patients treated at the early stages but strikingly no therapeutic efficacy at all when therapy was given at a slightly later stage. Cytokine combination therapy exhibits more focused and enhanced therapeutic response even when microglia depletion therapy already fails. It was further revealed that the optimal combination depends on the molecular profile of individual patients, suggesting the need for patient stratification and personalized treatment. These results, obtained solely by observing the *in silico* dynamics of the glioma microenvironment with no fitting to experimental/clinical data, reflect many characteristics of human glioma development and imply new venues for treating tumors via selective targeting of microenvironmental components.

## Author Summary

Tumor cells do not develop in isolation, but co-evolve with stromal cells via an array of soluble mediators. Here we report a model to integrate prior biochemical data and construct a glioma microenvironment *in silico*, which comprises 5 types of cells, 15 cytokines, and 69 signaling pathways. We observed a transition of the cytokine network from the microenvironmentally controlled, paracrine-based regulatory mechanism to the self-sustained, autocrine-dominant malignant state. A microglia-depletion therapy and a cytokine combination therapy were then designed and show significant efficacy on virtual patients. However, the optimal response depends on both the time the therapy is given and the molecular profiles of individual patients, suggesting the need for informative diagnosis and personalized treatment. These results, obtained solely by observing *in silico* tumor dynamics with no fitting to experimental/clinical data, reflect many characteristics of human glioma development and suggest new venues for anti-tumor treatment by selectively targeting microenvironmental components.

## Introduction

Tumor cells and stromal cells actively “talk” to each other via an array of soluble signaling molecules, leading to co-evolution of the tumor and its microenvironment [1], [2], [3], [4], [5]. This also implies that the tumor microenvironment itself is a critical aspect of disease mechanism and that the microenvironmental components, including cells and soluble mediators, may represent a new set of targets for anti-tumor therapy [3], [6], [7], [8], [9]. However, due to the inherent heterogeneity of the tumor microenvironment and the complexity of the cell-cell communication network, it remains poorly understood at the systems level how these cells and their communication network collectively shape a heterogeneous tumor microenvironment and modulate tumorigenesis and metastasis. Conventional approaches that examine one or two selected pathways are incapable of fully assessing complex signaling networks and recapitulate the dynamics of the tumor microenvironment, and often result in contradictory conclusions. Thus, a systems approach that examines various cell types and the associated intercellular signaling networks in the tumor microenvironment is highly desired.

In this work we choose to study the dynamics of glioblastoma multiforme (GBM) development. GBM is one of the most malignant brain tumors, with conventional therapies against “common” oncogenic targets usually ineffective due in part to the high degree of tumor heterogeneity. Astrocytes, microglia, and infiltrating immune cells actively interact with glioma and glioma stem cells via complex intercellular signaling networks mediated by an array of soluble signaling molecules, e.g., cytokines, growth factors, and neuropoientins [10]. All these collectively shape a tumor microenvironment that could be distinct from one patient to another. Despite substantial research efforts and significant advances in cancer therapeutics, human GBM remains the most aggressive and lethal brain tumor in humans. In addition to inter-tumoral and inter-patient heterogeneity, GBM also exhibits significant intra-tumoral heterogeneity down to the single-cell level [11], [12]. First, glioma cells originate from a variety of dynamically evolving progenitor cells [13]. It has been demonstrated that GBM cells demarcated by the neural stem cell marker CD133 exhibit much enhanced competencies for self-renewal and tumor initiation [14], [15]. Recent studies have also shown instances in which CD133-negative cells were able to generate the same outcomes [16], [17], [18], [19]. Second, glioma cells constantly interact with a variety of stromal cells. There is evidence that glioma cells acquire the ability to recruit and subvert their untransformed neighbor microglia into active collaborators to facilitate tumorigenesis. Direct correlation has been reported between the grade of glioma and the level of resident tumor microglia [20], suggesting the mutual paracrine stimulation between microglial cells and glioma cells [21], [22], [23], [24]. Microglial cells recruited by glioma can promote tumor growth [25], [26], [27], dictated by paracrine loops responsible for glioma initiation and progression (e.g., IL-6, IL-10, TGF-β, prostaglandins, G-CSF, and GM-CSF, and growth factors such as EGF, VEGF, HGF, and SCF). The crosstalk between activated astroglial and glioma cells has also been documented, although the mechanism of their interactions has not been full revealed. For example, astroglial cells produce IL-1β [28], [29] that promotes cell proliferation [30], [31], [32] and tumor angiogenesis [33], [34], [35]. Upon stimulation by the autocrine IL-1β these cells further secrete TNF-α and IL-6 [36], [37], [38]. The former was found to increase VEGF [39], EGF receptor [40], and MMP-9 [41] expression in glioma cells, suggesting that astroglia-produced cytokines may influence all the three most critical aspects of glioma cell survival: angiogenesis (VEGF), proliferation (EGFR), and migration (MMP-9).

*In silico* models of tumor microenvironment integrate information about the biological context in which cancers develop, and thus represent a multi-scale consideration of oncogenesis as it occurs within somatic tissues [42], [43]. Multiple factors involved in the development of an intrinsically complex tumor microenvironment have been studied including extracellular biomolecules, a spatially intricate and dynamic vasculature, and the immune system. Thus far, these models can be broadly divided into ‘continuum’ models, and discrete or ‘agent-based’ models as summarized in a review by Price and coauthors [43]. The latter describe the dynamics of individual interacting units, such as cancer cells, in small confined space; the former can be applied to a large tissue scale where agent-based modeling is computationally prohibitive. However, none of these methods have been integrated with a large cell-cell communication network in a complex tumor microenvironment. Herein we integrate all the intercellular signaling pathways known to date for human glioblastoma and generate a dynamic cell-cell communication network associated with the glioma microenvironment. Then we apply evolutionary population dynamics and the Hill functions to interrogate this intercellular signaling network and execute an *in silico* tumor microenvironment development. The observed results reveal a profound influence of the microenvironmental cues on tumor initiation and growth, and suggest new venues for glioblastoma treatment by targeting cells or soluble mediators in the tumor microenvironment.

## Results

### Constructing the intercellular signaling network of the glioblastoma microenvironment

**Figure 1:**
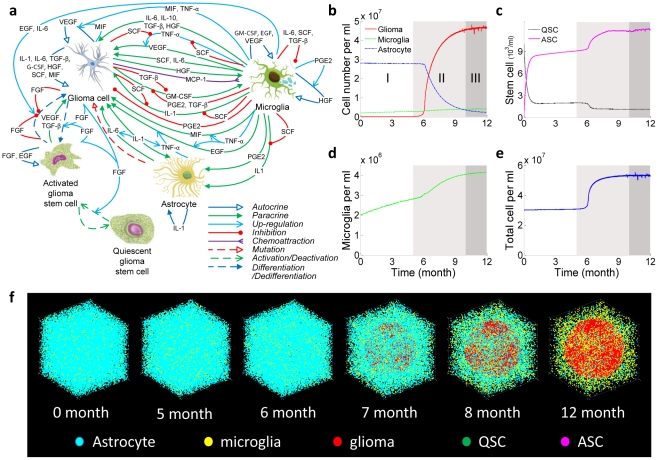
Stochastic population dynamics of glioma cells, glioma stem cells, astrocytes, and microglial cells. (**a**) Schematic representation of the intercellular signaling network in GBM. The network comprises 5 types of cells and a panel of 15 cytokines. The processes involving cytokine or chemokine mediation are described by solid lines, while the other processes representing changes of cell states are depicted by dashed lines. A detailed description of the ODEs and parameter settings are in Supporting Text S1. (**b**) One-year evolution of five types of cells showing three distinct phases: pre-tumor phase (I), rapid expansion phase (II), and malignant phase (III). (**c**) Dynamics of stem cell activation. (**d**) Dynamics of microglia cells. (**e**) Temporal change of total cell concentration. (**f**) Snapshots of temporal progression of tumor from a 3D Monte Carlo simulation. Supporting Video S1 is the complete video showing the one-year evolution. QSC, quiescent stem-like cell. ASC, activated stem-like cell.

Although much is known about the identities and biochemical activities of signaling molecules in the glioma microenvironment [1], [2], [3], [4], [5], [44], [45], how these mediators coordinate and function collectively at the systems level to regulate tumor development is insufficiently understood. Here we first constructed an intercellular signaling network by incorporating all the autocrine/paracrine pathways known for human glioblastoma, as shown in the diagram of Fig. 1a. Five types of cells – quiescent and activated glioma initiating/progenitor cells, glioma cells, and astroglial and microglial cells – and a panel of 15 growth factors/cytokines/chemokines were included in the signaling network. Then we derived a quantitative model using stochastic population dynamics and the Hill functions. First, a basic population dynamic equation was employed to compute the growth rate of five cell types as a function of their proliferation rate, decay(apoptosis) rate, the rate of formation via direct mutation, the rate of formation via differentiation of their stem/progenitor cells, and the rate of de-differentiation. Second, the temporal growth rate of each cell type is also modulated by soluble signaling mediators present in the tumor microenvironment; this process is quantitatively described by the Hill functions. All differential equations are described in Supporting Text S1 and the initial settings of all parameters are detailed in Supporting Table S1. As an example, we present here the procedure on how to construct the model for glioma cell population. It has been suggested that glioma can originate from cells at multiple differentiation stages during glial cell development, whereas the progenitor cells appear to be more susceptible to neoplastic transformation compared with mature glial cells [46], [47]. Cytokine signalings, including IL-1, IL-6, IL-10, TGF-β, EGF, VEGF, HGF, G-CSF, SCF, and MIF, participate in the mechanism of promoting GBM growth. PGE2 can transiently prevent glioma cell proliferation *in vitro*. EGF, FGF, and MIF are predominantly survival factors for GBM cells. To re-illustrate the underlying physics of this model, we show the population dynamics for glioma cells as in equation 1, which integrate all the above signalings:
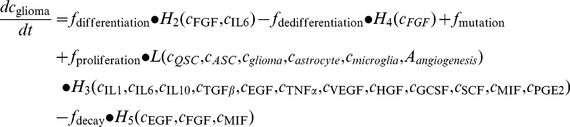
(1)where *c*_cell/cytoine_ is the concentration of cell/cytokine, *f* is the basal rate function, *H*_1_, *H*_2_, *H*_3_, *H*_4_, and *H*_5_ are Hill functions, *L* is a logistic function, and *A*_angiogenesis_ is defined as the angiogenesis factor. Similarly, the same algorithm was applied to derive population dynamics equations for other cells. More details may be found in Method and the sections 1&2 in Supporting Text S1.

The change of cytokines associated with tumor microenvironment development is described as the production and consumption by all the cells and modulation by other cytokines as revealed by prior experiments. For example, glioma stem cells, glioma cells, and microglial cells secrete substantial amounts of VEGF. MIF and TNF-α have been observed to induce a significant dose-dependent increase of VEGF. The dynamics of VEGF is thus governed by a differential equation (Eq. 10) related to these cells and soluble mediators:
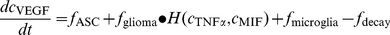
(2)where *f* is the basal secretion/decay rate function and *H* is the Hill function. In the end, the temporal rate of growth and death of each cell population or the rate of production and decay of each cytokine is expressed as an ODE; a set of 20 inter-coupled ODEs were constructed to interrogate the dynamics of intercellular signaling network in a glioma microenvironment. To capture the stochastic nature of cell dynamics and cytokine signaling, we applied truncated Gaussian white noise, Poisson white noise, and bounded noise to describe the stochastic perturbation to production/regulation rate constants, recruitment rate, and proliferation/mutation/differentiation rates, respectively. Supporting Tables S1 and S2 and Methods give a complete description of all the signaling processes and summarize the input values for all differential equations.

### Dynamics of glioma cells, glioma stem cells, astrocytes, and microglial cells

We performed an *in silico* stochastic study of glioma microenvironment development in a 1-ml control volume over a period of 12 months and observed a non-linear, synergistic co-evolution of all five cell types (Fig. 1b–f). The dynamics of glioma cells (GC) exhibit three distinct phases (Fig. 1b): the pre-tumor phase (1–5 months), the rapid expansion phase (6–10 months), and the malignant phase that corresponds to semi-steady high-grade glioblastoma (11–12 months). The starting cell populations are astrocyte (2.8×10^7^/ml), microglia (2×10^6^/ml), and quiescent stem cells (QSC) (1×10^4^/ml). The initial conditions only change the quantitative timeline of the dynamics but would not affect the general trends observed in our model that properly reflect the dynamics of human glioma (see Supporting Fig. S2). The number of glioma cells at t = 0 is zero, and glioma cells develop via either neoplastic transformation of normal astrocyte or differentiation of glioma stem cells. The initial rate constants (time = 0) are derived from literature reports [48], [49], and become gradually subjected to the modulation by soluble factors (cytokines and growth factors). We observed that glioma stem cells are the major cell sources for glioma formation. At the early stage, QSCs upon stimulation are rapidly activated into activated stem cells (ASC) via a reversible process conferring self-renewal capability (Fig. 1c). This step proceeds to completion within the first month. Then both QSCs and ASCs stay at a relatively steady state over the next four months before ASCs further differentiate into glioma cells in a stochastic manner. Despite the rapid lineage conversion of stem cells occurring as early as in the first month, glioma cells remain at a silent state with cell density way below the clinically detectable threshold. (For all the experiments shown here, the threshold for detecting glioma in the clinic is assumed to be 1×10^6^/ml, which is in agreement with the data from clinical studies [50].) During the growth of glioma cells within the space that astrocytes occupy, astrocytes strive to maintain their abundance as well as their functions until they are displaced by the glioma cells in the late stage. The number of microglial cells follows a steady increase all the way from the pre-tumor to the malignant stage, with a small kink occurring at the onset of rapid tumor expansion (Fig. 1d).

Although no “clinical” signs are observed at the first phase, the imperceptible changes occurring in the tumor microenvironment silently accumulate tumorigenic signals and eventually result in a switch of fitness dominance between astrocyte and glioma. The glioma cells acquire competitive advantages and are primed to rapid growth within a month to reach the diagnostic threshold (∼1×10^6^/ml). This unique behavior is consistent with glioblastoma development observed in animal models [17]. It was assumed that after rapid expansion glioma cells follow a typical exponential growth mode in the next month until reaching a tumor cell concentration (∼1.5×10^7^/ml), and then gradually turn into a slow growth phase dictated by the logistic growth model [51], [52]. The astrocyte population shrinks due to competitive selection pressure exerted by a microenvironment unfavorable to astrocyte proliferation or favorable to astrocyte apoptosis that decreases the fitness advantages over time and eventually causes the loss of dominance. We examined the contribution of direct mutation of astrocyte and the differentiation of glioma stem cells to glioma growth. We observed that neoplastic transformation of astrocytes directly to glioma cells does result in the formation of small numbers of glioma cells in the pre-cancer phase, but contributes little to tumor development in rapid growth and expansion phases (see Supporting Fig. S1). The total cell concentration experienced a significant expansion during the seventh month, suggesting a density-gradient-driven potential for the glioma cells to invade neighboring tissues (Fig. 1e). The total cell density we observed in the tumor microenvironment is higher than that in normal tissue, which is quantitatively consistent with the results obtained using tissue histology examinations [53], [54], [55]. A three-dimensional (3D) stochastic simulation (Fig. 1f) shows that the evolution of all the cell types and the time course are consistent with clinical glioblastoma development.

### Cytokine dynamics and interaction

**Figure 2:**
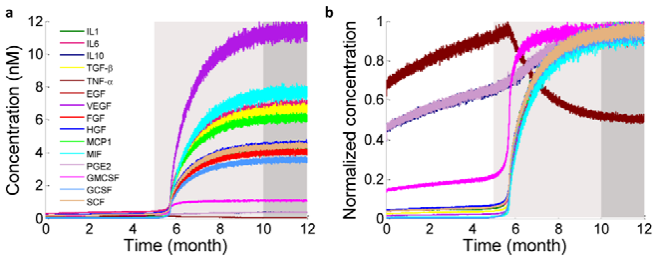
Cytokine dynamics. (**a**) Change of concentrations for all 15 cytokines in the microenvironment over a period of one year. (**b**) Normalized cytokine concentration change over a period of one year. It shows three types of cytokine dynamics based upon the temporal traces. TNF-α peaks at the end of phase I. IL10 and PGE2 show constant increase regardless the growth phases of glioma cells. Other cytokines are apparently correlated to the three-phase growth dynamics.

Cytokine dynamics also exhibit multi-stage non-linear characteristics (Fig. 2a). Activated microglial cells were found to be an important source of cytokines in the early stage, yielding a steady increase of cytokine concentrations prior to the emergence of tumor. These cytokines participate in the modulation of rapid glioma cell expansion in the later stage, suggesting that microglial cells may play an important role in tumor initiation by priming glioma cells at very low concentrations. Glioma cells also secrete paracrine signaling factors that promote the proliferation and migration of microglia, and thus in turn benefit from the increase of microglia cells that reside in the vicinity of the glioma growth front. The normalized dynamics curves (Fig. 2b) show that 15 cytokines fall into three categories according to their time traces. TNF-α peaks at the end of the first phase, then gradually decreases presumably due to the consumption by glioma cells (e.g., rebind to TNF receptors and trigger the secondary signaling cascades). IL10 and PGE2 show a monotonic increase across all the three phases. All the other cytokines exhibit a rapid concentration increase in the second phase and reach a quasi-steady state correlated with the glioma population dynamics.

### Therapy targeting the cells in the tumor microenvironment: microglia depletion

We first designed a novel therapy by targeting the cellular components of the tumor microenvironment. According to cell population dynamics (Fig. 1), microglial cells produce an array of cytokines that often prime glioma cells to predispose them to rapid population expansion in the sixth month, and thus function as a tumor-promoting factor in the tumor microenvironment. Therefore, we designed a cell-targeting therapy that eliminates microglial cells in the tumor microenvironment.

**Figure 3:**
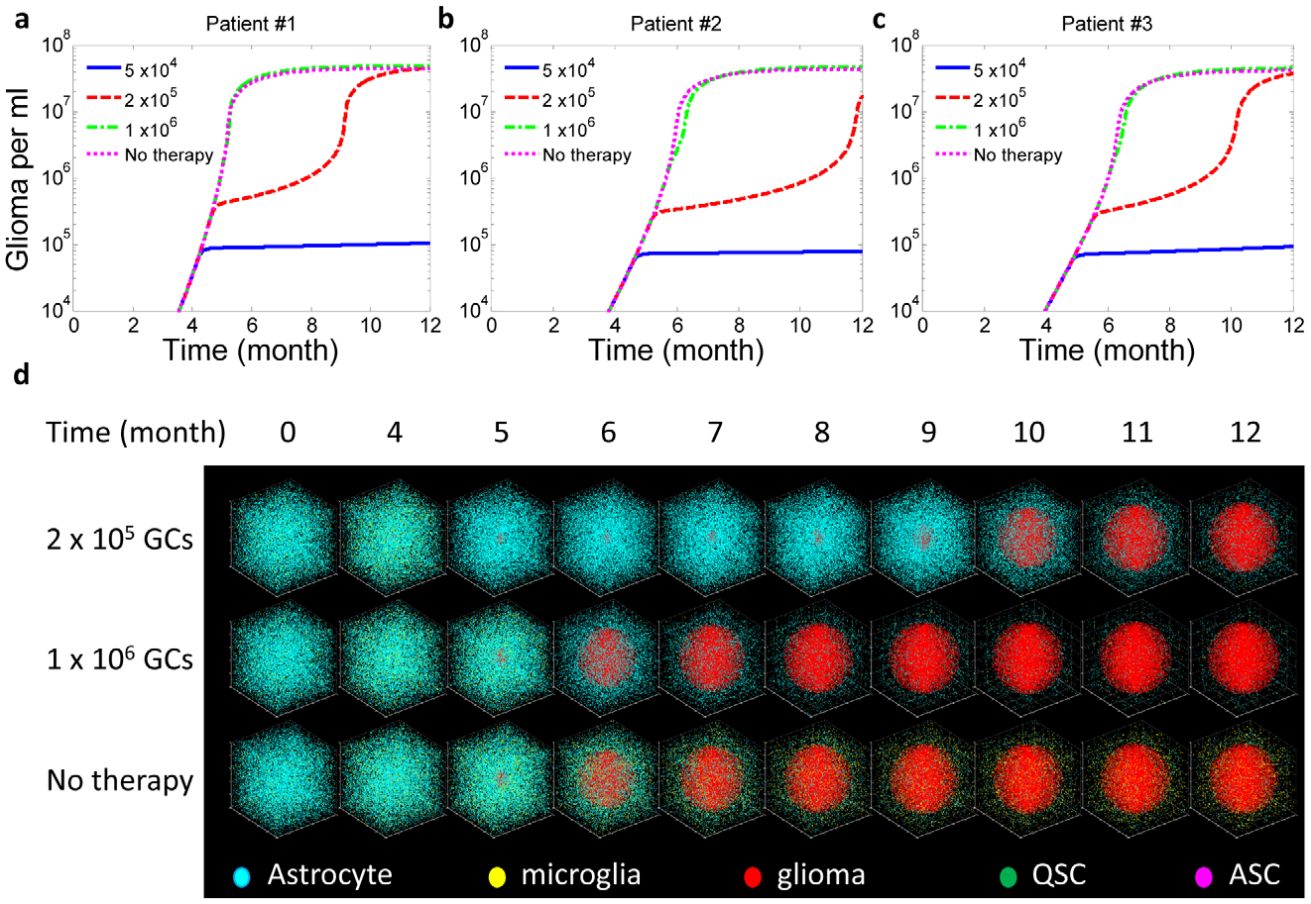
Microglia depletion therapy. This therapy was given to three randomly designed virtual patients (Supporting Table S5) and administered at different stages corresponding to glioma cell (GC) concentration ∼5×10^4^/ml, 2×10^5^/ml, and 1×10^6^/ml, respectively. (**a**) Response of patient 1 to therapies given at different stages. (**b**) Response of patient 2 to therapies given at different stages. (**c**) Response of patient 3 to therapies given at different stages. (**d**) Snapshots of a 3D simulation showing the evolution of tumor microenvironment in patient 1 in response to microglia depletion therapy. Supporting Video S2 shows the full video.

This therapy is realized by arbitrarily increasing the apoptotic rate of microglia by 10 times at the early, middle, and middle to late stages with the corresponding glioma cell density at 5×10^4^/ml, 2×10^5^/ml, or 1×10^6^/ml, respectively. To examine the applicability of this therapy to patients with different biomolecular background and assess the effect of inter-patient heterogeneity on therapeutic response, three virtual patients with different profiles of initial parameters (cytokine production rate, receptor expression level, etc.) within the ranges reported in the literature [56] (Supporting Table S5) were treated using the same microglia depletion therapy at three different stages. The results are compared as shown in Fig. 3a–c. Two interesting features were observed in the microglia depletion therapy experiments. First, all patients responded in a similar manner although the length of therapeutic benefit and the recurrence time varied from one patient to the other. Second, the efficacy strongly depends on how early the treatment was given to the patients (Fig. 3d). All the patients treated at the early stage when glioma cell density (∼5×10^4^/ml) is far below the threshold for clinical tumor detection (1×10^6^/ml) showed no recurrence within the time of simulation. Treatment given at the early to middle stage (glioma cell density ∼2×10^5^/ml) postpones the rapid tumor growth phase by two to four months and does give the patient therapeutic benefit. Patients treated right as the clinical sign emerges (glioma cell density∼1×10^6^/ml) did not respond at all in terms of glioma growth rate, suggesting that tumor cells have been fully primed and become self-sustained with no need of paracrine signaling to drive glioma cell proliferation.

These results, obtained by unbiased integration of basic biochemical parameters and cell signaling processes, were found to appropriately reflect clinical and experimental observations. There is a consensus that activated microglia promote glioma growth and promotion, which is consistent with our *in silico* glioma development experiments [20], [39], [40], [41]. Recently, an animal model study indicated that clonal cooperation between different mutant cells can lead to tumor formation, whereas any single-cell type alone cannot develop into tumor [57]. What is more interesting is that the second clone, once activated by the first clone presumably through cytokine signaling, becomes fully self-sustained and develops into tumor without the presence of the first clone, which is strikingly similar to the glioma-microglia interaction observed in our model, and thus may share commonalities in molecular and cellular mechanisms. Our study suggests that cells in the tumor microenvironment can be good targets for therapeutic intervention or control of tumor progression, pointing to new venues for anti-tumor drug design and development.

### Combination therapy targeting multiple cytokines

The results of microglia-depletion therapy indicate that patients do not show significant responses unless they are diagnosed at the very early stage – the time when no clinically detectable tumors have been formed. Thus, we turn to assess the possibility of combination therapy that directly targets a number of key cytokine signaling pathways, which is anticipated to give more focused and potent therapeutic effects.

**Figure 4:**
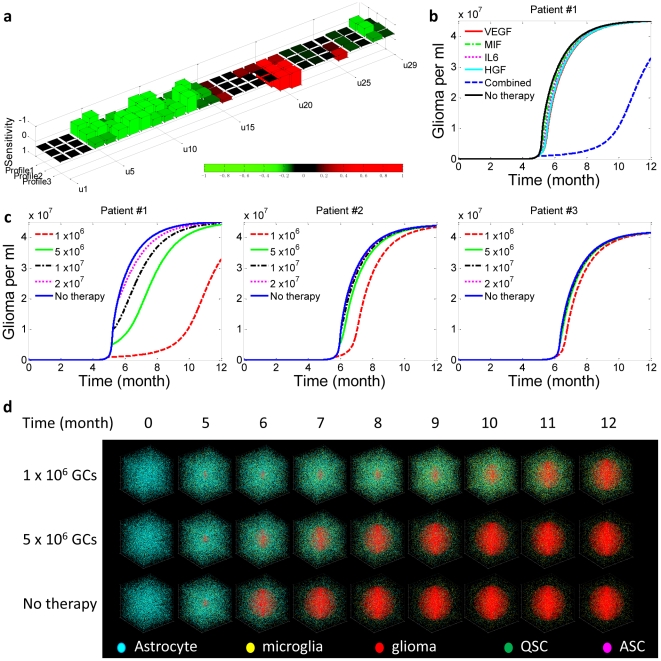
Cytokine combination therapy. This was given to the same three virtual patients (Supporting Table S5) and administered at different stages, corresponding to glioma cell (GC) concentration ∼1×10^6^/ml, 5×10^6^/ml, 1×10^7^/ml, and 2×10^7^/ml, respectively. (**a**) Sensitivity analyses reveal the pro-tumorigenic potential of each cytokine in the examined tumor microenvironment. Supporting Table S7 summarizes all the parameters (u1–u29), surface receptor expression level. (**b**) Comparison of therapeutic efficacy between single-target and combination therapies. The combination therapy results in a striking synergistic effect to suppress tumor progression whereas any single target treatment does not show appreciable benefit. (**c**) Reponses of three patients to the same combination therapy, which is tailored to give optimum response for patient 1 based upon sensitivity analysis. (**d**) Snapshots showing tumor development in patient 1 with or without combination therapy. The therapy is give at a single dose when the glioma cell density reaches 1×10^6^/ml or 5×10^6^/ml. Supporting Video S3 is the complete video showing the one-year evolution.

Due to inter-tumoral heterogeneity, the best therapeutic regimen must be an individually tailored combination of inhibitors that act on selected cytokines or their receptors optimized for the patient. We performed a sensitivity analysis to assess the tumorigenic potential of each cytokine and find the primary targets that, once subjected to blockade or promotion, exhibit the most effective responses in therapeutic intervention. The Methods and section 3 in Supporting Text S1 describe the details of this analysis. Basically, it measures the length of time taken by glioma cells to grow from the threshold concentration (e.g., 1×10^6^/ml) to an objective concentration (e.g., 1.5×10^7^/ml) reflecting the survival time of a patient after the therapy is given. Twenty-nine tests, each perturbing a cytokine production rate or a cytokine receptor expression level, were performed to give the sensitivity factor of each cytokine or its receptor with respect to patient survival probability. According to the results, forced activation of a signaling pathway with a positive sensitivity factor is expected to promote patient survival, and vice versa. Individualized combination therapy is designed by enhancing the signaling processes of cytokines with the largest “positive” sensitivity factors and inhibiting those with the largest “negative” sensitivity factors. To test this therapy, the same virtual patients (patients 1, 2, and 3) that were randomly designed for microglia depletion experiments are examined here to generate sensitivity factor profiles for every patient (Fig. 4a). Next we designed a four-cytokine combination therapy (VEGF, MIF, IL6, and HGF) optimized for patient 1, and all the patients were given the same treatment for comparison.

First, we compared single-target therapy and combination therapy that are administered at the time of glioma cell density∼1×10^6^/ml (Fig. 4b and Supporting Fig. S3). Although each of the four cytokines has a large negative sensitivity factor for promoting tumorigenesis, therapies that inhibit only one of these cytokines can hardly alter the time course of tumor progress, due to the homeostatic robustness of the cytokine network and the resulting intrinsic resistance to perturbation. To overcome this issue, we further applied to virtual patient 1 a combination treatment that simultaneously inhibits all four cytokines, and we observed substantial therapeutic responses that cannot be simply explained by the additive effect (Supporting Fig. S4). Second, the same therapy was given to patients 2 and 3, but did not yield positive therapeutic responses (Fig. 4c and Supporting Fig. S5); patient 2 exhibited a modest benefit by one month and patient 3 almost did not respond at all. Figure 4d shows the results of a 3D stochastic simulation of cell population dynamics in response to combination therapy administered at different times. Considering that these treatments were administered at a middle to late stage when clinically detectable tumors had already developed, we conclude that the combination therapy tailored to match individual patients is more focused and can give better therapeutic benefit even when microglia depletion therapy fails in the middle to late stages, highlighting the critical need for molecular diagnosis and patient stratification prior to the design of a combination therapy that targets the tumor microenvironment.

## Discussion

To the best of our knowledge, this is the first study that attempts to integrate a variety of cells and their intercellular signaling pathways into a cell-cell communication network and assess how this network controls tumor initiation and progression at the systems level. Through *in silico* experimentation of tumor microenvironment development, the dynamics of cells and cytokines correctly reflects general trends of tumorigenesis observed experimentally or clinically [17], [51], [58]. We also discovered interesting phenomena that can be seen only at the systems level and are often masked in conventional tumor biology studies.

First, the cell population dynamics obtained using a set of coupled differential equations based upon population dynamics and the Monte Carlo method yield the full time courses of all five cell types. Although significant inter-patient heterogeneity has been observed, the time courses of glioma microenvironment development for all virtual patients we encountered do share common characteristics and all exhibit three-phase non-linear evolution dynamics. For example, all patients experience the pre-tumor phase; the mutual paracrine stimulation between microglial cell and glioma cell results in the continued growth of microglia. These results, obtained via *in silico* experimentation without fitting or optimization to any specific clinical or experimental data, were found to well reflect the general mechanisms of glioma development [17], [20].

Second, soluble signaling proteins, e.g. cytokines, are the key components mediating the cell-cell communication network in a tumor microenvironment. We successfully integrated 15 cytokines in 69 paracrine/autocrine pathways in the cell population dynamics model. We further examined relative weight factors for all the paracrine/autocrine loops associated with tumor development. This study provides new insights into tumor microenvironment development and suggests that therapies targeting the cytokine-mediated intercellular signaling network in a tumor microenvironment need to be personalized.

Third, we designed a microglia depletion therapy by adding a virtual drug in the tumor to increase the microglia apoptosis rate. The observation from *in silico* experimentation indicates that this therapy shows some efficacy only when patients are treated at very early stages, which is consistent with the general outcomes of anti-cancer treatment, but provides a new mechanism to explain the therapeutic resistance observed in the clinic. The ineffectiveness of microglia-targeted therapy in the middle to late phases indicates the emergence of an autocrine-dominant, self-propelled glioma proliferation. Then, we moved to look for another therapy that directly targets multiple key cytokines to assess the possibility of treating glioma in the middle to late stages. It turns out a more focused combination therapy can suppress tumor growth at the middle stage when the tumor becomes clinically detectable and microglia-depletion therapy is ineffective. Further study on virtual patients reveals inter-patient heterogeneity in response to the same combination therapy, and highlights the importance of designing therapy individually tailored to the patient's tumor microenvironment. While current anti-cancer drugs mostly target tumor cells, this study indicates the possibility and quantitatively assessed the effectiveness of new therapies that target cellular or molecular components of the tumor microenvironment, pointing to completely new venues for tumor control and treatment.

In the end, a model as reported herein may serve as a tool to integrate clinical data obtained from informative molecular diagnosis of patients, predict the dynamics of tumor progression, and aid the design of personalized therapy. The technologies for such informative diagnosis are anticipated (1) to measure both tumor cells and a variety of cells in a tumor microenvironment, and (2) to analyze cytokine secretion profiles at the single-cell level such that a cytokine-mediated cell-cell communication network can be re-constructed for any individual patient. Currently, such technologies are not yet available in the clinic, but there have been significant research efforts in the past years that aim to develop single-cell proteomics technologies and clinical microchips for informative diagnosis of complex diseases including cancer [59], [60], [61], [62],[63]. In the future, integration of such technologies and the model described here can turn into a powerful clinical tool to diagnose the tumor microenvironment and the associated intercellular signaling network in individual patients and truly enable personalized therapy by selective targeting of the tumor microenvironment.

## Methods

### Algorithm

While the microenvironment exerts a significant selective pressure on the tumor, the tumor cells persistently reshape their microenvironment to synergistically support the growth and spread of the tumor. The dynamically changing levels of signaling molecules that rewire tumor-stromal interactions along with tumor progression will provide insights into the mechanisms of disease development.

### Assumptions and model construction

Since this work is focused on predicting tumor time course evolution, the model is based, for simplicity, on a well-mixed species system. Five types of cells (quiescent and activated glioma stem/progenitor cells, glioma cells, astrocytes, and microglial cells) and 15 growth factors/cytokines/chemokines are integrated in this model. We assume that the species included in the model evolve independently of species excluded from the model (oligodendrocyte, etc.). In the end, we present the intercellular signaling network as a set of coupled ordinary differential equations in terms of population dynamics. The rates of change of cells are expressed by the conversion rate, proliferation rate, and decay rate (see Supporting Text S1).

A set of coupled stochastic ordinary differential equations describing the co-evolution of tumor microenvironment is constructed using population dynamics and stochastic dynamics. The basic mathematical model is based on continuous logistic proliferation and discrete event type fluctuation, and can be described as
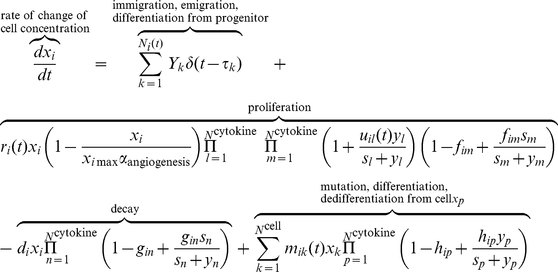
(3)

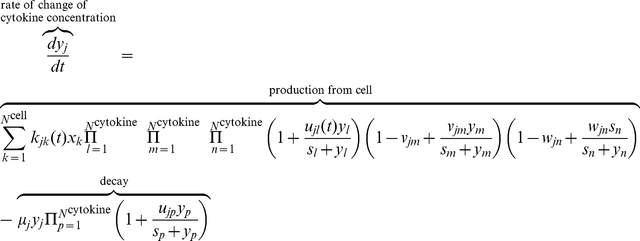
(4)

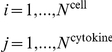
where the first term of the right-hand side of Eq. (3) is the stochastic representation of discrete event–type fluctuation, including immigration, emigration, and production from progenitor. 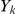 is the magnitude of the *k* ^th^ discrete event, i.e., the number of cells increasing (decreasing) at time point 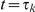. 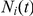 denotes a non-homogeneous Poisson counting process with arrival rate function 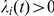 (i.e., the number of events per unit time) and gives the number of events that arrive in the time interval 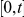. The second term indicates the logistic proliferation of cell 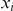 with a basic rate function 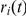, which can be up-regulated by cytokine 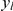 and inhibited by 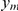. Parameter 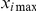 is the saturating concentration factor, whereas 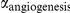 is the angiogenesis factor. The initial exponential growth will slow down and the cell concentration level 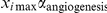 is approached slowly in the late time. The third term is the decay due to natural lifespan that can be regulated by cytokine 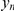. The last term describes the mutation/differentiation/dedifferentiation from cell 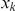 under stimulation of cytokine 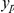. The first term on the right hand side of Eq. (4) quantifies the production of cytokine 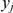 from cell 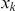 with basic secretion rate function 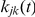, and the secretion is stimulated by cytokine 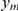 and inhibited by 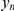. The last term is the decay term with half-life 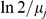, and can be regulated in the presence of cytokine 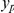.

To further assess how fluctuations in biological processes reflect the random nature and affect the performance of the system, we introduce the following stochastic process interpretation of the rate parameters:
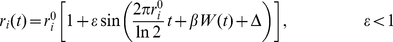
(5)

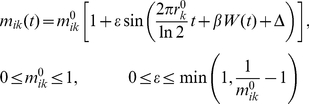
(6)

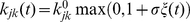
(7)

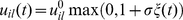
(8)

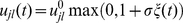
(9)

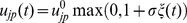
(10)where *W*(*t*) is a standard Wiener process, and *ξ*(*t*) is a zero-mean Gaussian white noise with unit intensity. The sections 1&2 in Supporting Text S1 give a full set of deterministic ordinary differential equations (ODE) and detailed explanations of stochastic description.

The stochastic dynamics are studied using Monte Carlo simulations. The corresponding time series of the species concentration are obtained by integrating these differential equations numerically using the fourth-order Runge-Kutta scheme or the fifth-order Dormand-Prince method.

### Model calibration

The parameters are assigned in the range over which the model output most closely matches experimental observation (Supporting Table S1). Although we calibrate the model with data from the literature, the model parameters can easily be changed to patient-specific clinical parameters as needed.

### Sensitivity analysis

To systematically evaluate the influence of each cytokine on tumorigenesis rate, we conduct a sensitivity test, in which the sensitivity factor of cytokine *x_i_* can be calculated as
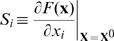
(11)where *F*(**x**) is the objective function (e.g., tumorigenesis time, cell density, cytokine concentration), and **x** ^0^ is the local parameter profile.

The results show marked effects of these cytokines on the development of glioma and suggest the possibility of designing therapeutic intervention by targeting cytokine signaling loops (both cytokine production and receptor expression level) (Supporting Fig. S6 and Table S6). The quantitative results are also found to be context specific; the exact time for observing tumor formation (1×10^6^ cells/ml) depends on the profile of all initial parameters for each virtual patient (Supporting Tables S3 and S4). The greater the difference between cytokine sensitivity factor landscapes, the greater is the inter-patient heterogeneity. In addition to the quantitative manifestation of inter-patient heterogeneity, sensitivity analysis also points to a venue to identify a cytokine profile that potentially can serve as a molecular signature for tumor sub-classification, and thus provides a means to stratify patients via their cytokine profiles and to design individualized treatment.

## Supporting Information

Figure S1 Contributions of ASC differentiation and asctrocyte mutation to glioma development.
(TIF)

Figure S2 Influence of initial conditions to tumorigenesis time.
(TIF)

Figure S3 Virtual therapy of patient #3 demonstrates the difference of therapeutic efficacy between single-targeted and combination-targeted.
(TIF)

Figure S4 Virtual therapies of two patients demonstrate the therapeutic efficacy of combination-targeted therapy.
(TIF)

Figure S5 Three patients are treated with the same protocol, which is personalized according to the cytokine secretion profile of patient #3.
(TIF)

Figure S6 Inter-patient heterogeneity was demonstrated by sensitivity analyses.
(TIF)

Table S1 Deterministic parameters.
(DOCX)

Table S2 Stochastic parameters.
(DOCX)

Table S3 Patients parameters for Figure S5(a).
(DOCX)

Table S4 Patients parameters for Figure S5(b).
(DOCX)

Table S5 Parameter profiles of three virtual patients for Figure 3 and 4.
(DOCX)

Table S6 The x-coordinate parameter panels for Figure S5(a).
(DOCX)

Table S7 The x-coordinate parameter panels for Figure 4(a) and Figure S5(b).
(DOCX)

Text S1 Section 1: Deterministic description of the intercellular signaling network. Section 2: Stochastic description of rate parameters. Section 3: Sensitivity analysis.
(PDF)

Video S1 Evolution of tumor microenvironment without therapy.
(MPG)

Video S2 Evolution of tumor microenvironment in patient #1 in response to microglia depletion therapy administered at glioma cell concentration 2×10^5^/ml.
(MPG)

Video S3 Evolution of tumor microenvironment in patient #1 with personalized combination therapy when the glioma cell density reaches 1×10^6^/ml.
(MPG)
