# Supplementary figures and images for: In silico Experimentation of Glioma Microenvironment Development and Anti-tumor Therapy
Source: PLoS Comput Biol. 2012 Feb 2;8(2):e1002355. doi: 10.1371/journal.pcbi.1002355 (PMC3271023; doi:10.1371/journal.pcbi.1002355)

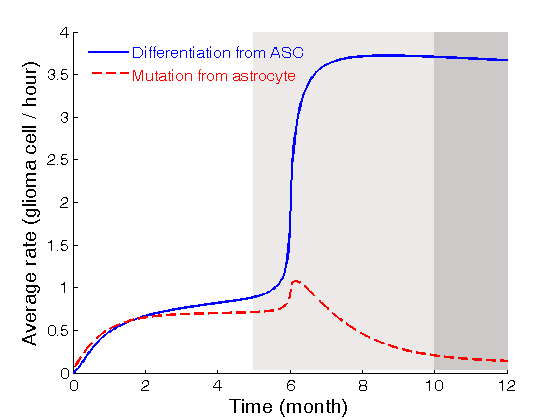

Supplement: Figure S1 — Contributions of ASC differentiation and asctrocyte mutation to glioma development. (TIF) [file pcbi.1002355.s001.tif]

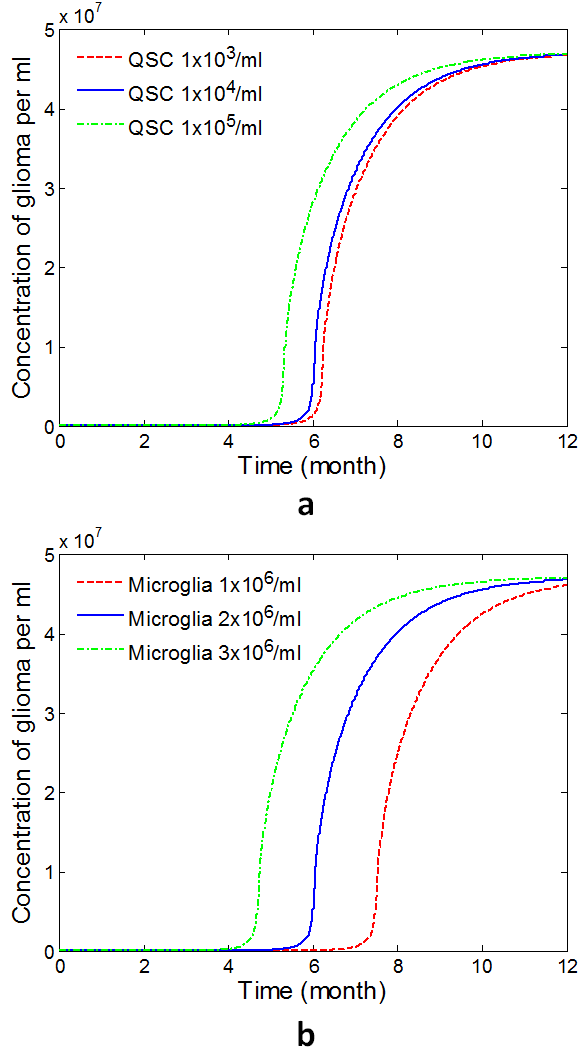

Supplement: Figure S2 — Influence of initial conditions to tumorigenesis time. (TIF) [file pcbi.1002355.s002.tif]

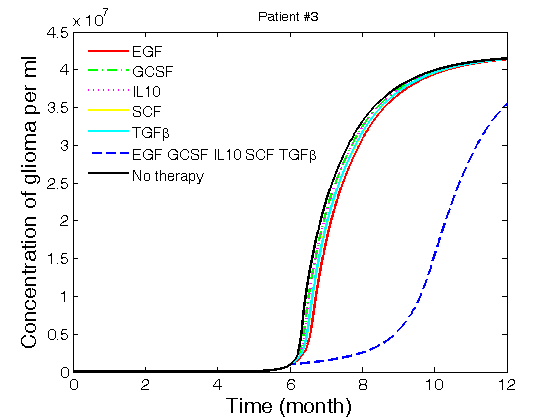

Supplement: Figure S3 — Virtual therapy of patient #3 demonstrates the difference of therapeutic efficacy between single-targeted and combination-targeted. (TIF) [file pcbi.1002355.s003.tif]

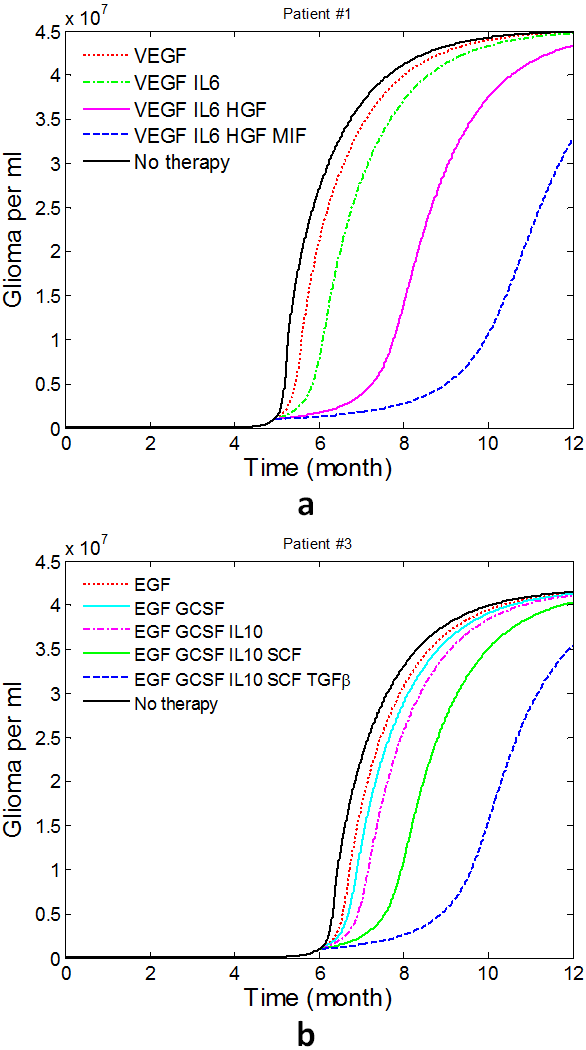

Supplement: Figure S4 — Virtual therapies of two patients demonstrate the therapeutic efficacy of combination-targeted therapy. (TIF) [file pcbi.1002355.s004.tif]

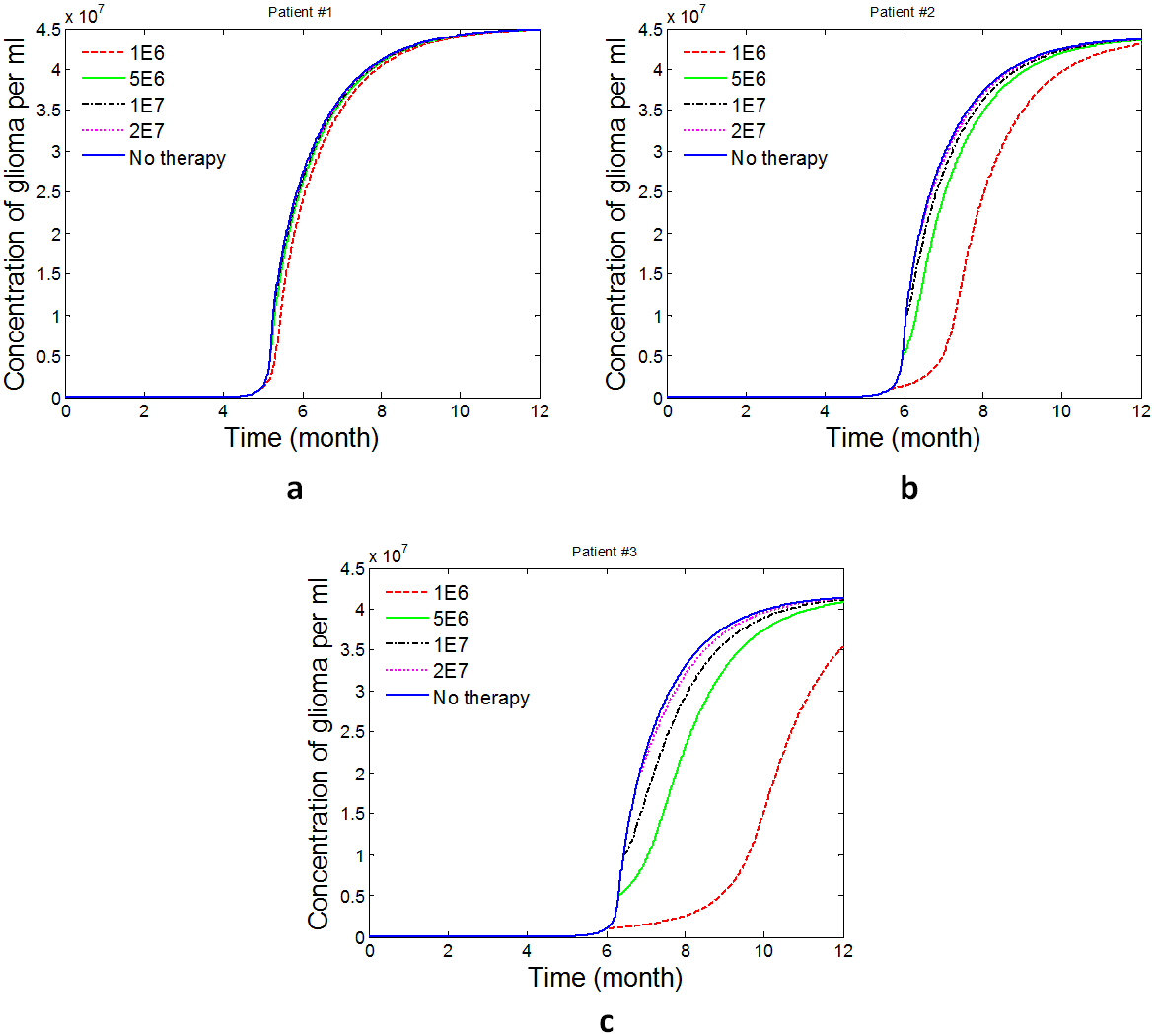

Supplement: Figure S5 — Three patients are treated with the same protocol, which is personalized according to the cytokine secretion profile of patient #3. (TIF) [file pcbi.1002355.s005.tif]

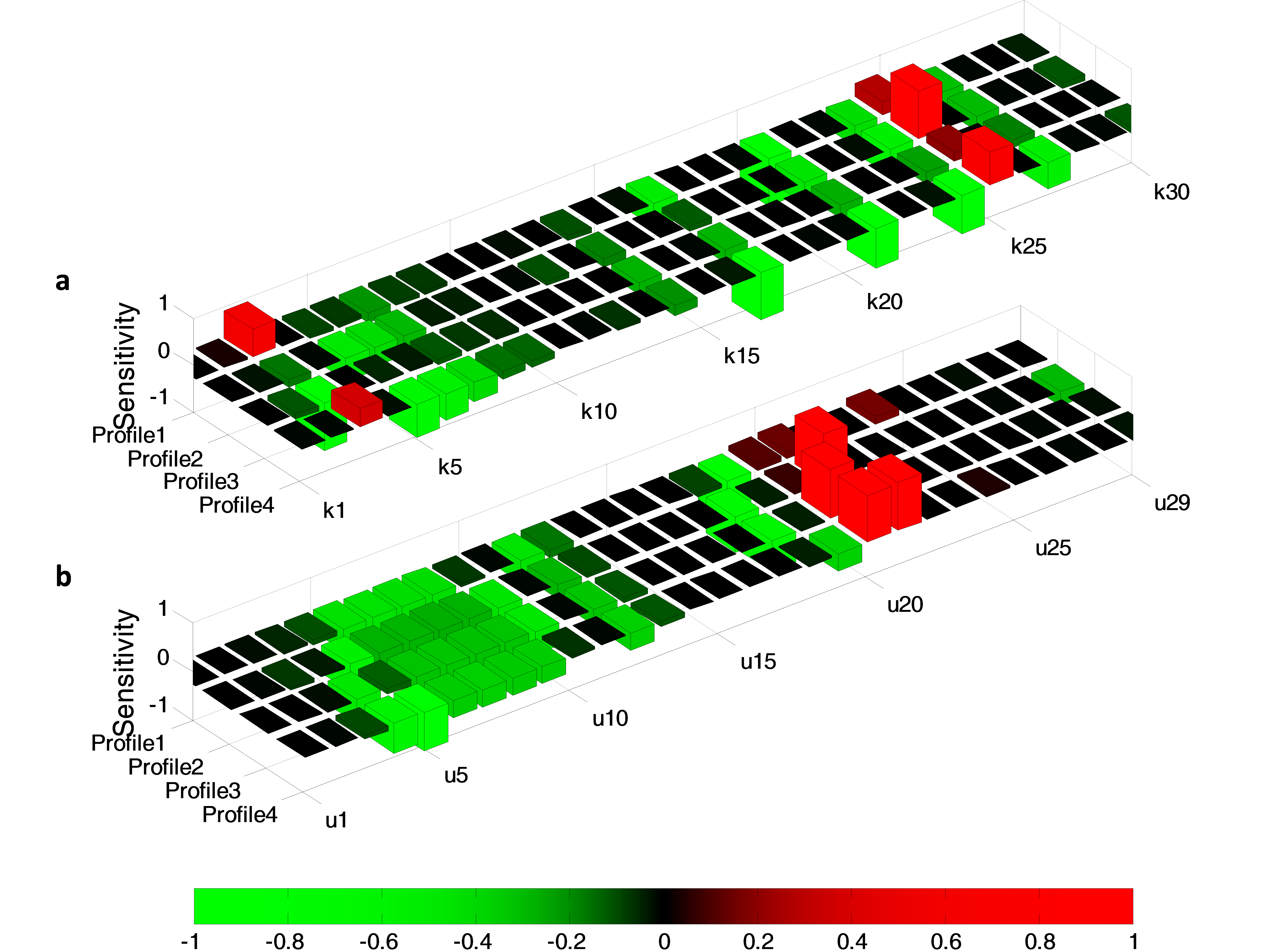

Supplement: Figure S6 — Inter-patient heterogeneity was demonstrated by sensitivity analyses. (TIF) [file pcbi.1002355.s006.tif]
