# Supplementary material for: In silico Experimentation of Glioma Microenvironment Development and Anti-tumor Therapy
Source: PLoS Comput Biol. 2012 Feb 2;8(2):e1002355. doi: 10.1371/journal.pcbi.1002355 (PMC3271023; doi:10.1371/journal.pcbi.1002355)
Supplement: Table S1 — Deterministic parameters. (DOCX) [file pcbi.1002355.s007.docx]

**Supporting Table S**1. Deterministic Parameters

| Variable | Description | Value | Dimension | Reference | Comment |
| --- | --- | --- | --- | --- | --- |
| *x*_QSC_ | Concentration of quiescent glioma stem like cell | 1E4 | ml^-1^ |  | Initial density |
| *x*_ASC_ | Concentration of activated glioma stem like cell | 0 | ml^-1^ |  | Initial density is zero |
| *x*_glioma_ | Concentration of glioma cell | 0 | ml^-1^ |  | Initial density |
| *x*_microglia_ | Concentration of microglia | 2E6 | ml^-1^ | [^122-125^](#_ENREF_122) | Initial density |
| *x*_astrocyte_ | Concentration of astrocyte | 2.8E7 | ml^-1^ |  | Initial density |
| *y*_IL1_ | Concentration of IL1 | 150 | pM | Estimated | Initial Concentration |
| *y*_IL6_ | Concentration of IL6 | 250 | pM | Estimated | Initial |
| *y*_IL10_ | Concentration of IL10 | 150 | pM | Estimated | Initial |
| *y*_TNFα_ | Concentration of TNFα | 80 | pM | Estimated | Initial |
| *y*_TGFβ_ | Concentration of TGFβ | 150 | pM | Estimated | Initial |
| *y*_EGF_ | Concentration of EGF | 150 | pM | Estimated | Initial |
| *y*_VEGF_ | Concentration of VEGF | 150 | pM | Estimated | Initial |
| *y*_FGF_ | Concentration of FGF | 0 | pM | Estimated | Initial |
| *y*_HGF_ | Concentration of HGF | 150 | pM | Estimated | Initial |
| *y*_MCP1_ | Concentration of MCP1 | 0 | pM | Estimated | Initial |
| *y*_COX2_ | Concentration of COX2 | 40 | pM | Estimated | Initial |
| *y*_PGE2_ | Concentration of PGE2 | 150 | pM | Estimated | Initial |
| *y*_GMCSF_ | Concentration of GMCSF | 150 | pM | Estimated | Initial |
| *y*_GCSF_ | Concentration of GCSF | 0 | pM | Estimated | Initial |
| *y*_SCF_ | Concentration of SCF | 150 | pM | Estimated | Initial |
| *c*_QSC_ | Rate of supply of quiescent glioma stem cell from normal neural stem cell | 1 | ml^-1^ h^-1^ | Estimated |  |
| *c*_microglia_ | Rate of replenishment of microglia from monocytes | 800 | ml^-1^ h^-1^ | [^57^](#_ENREF_57) |  |
| *c*_astrocyte_ | Rate of supply of astrocyte from progenitors | 2000 | ml^-1^ h^-1^ | Estimated |  |
| *r*_ASC_ | Average basal proliferation (including self-renewal and differentiation) rate of activated glioma stem cell obtained in the absence of population limitation and cytokine stimulus | 5E-4 | h^-1^ | [^131^](#_ENREF_131) | $\frac{\ln2}{\text{cell} \text{cycle} \text{period}}$ Cell cycle ~58 days |
| *r*_glioma_ | Average basal proliferation rate of glioma cell obtained in the absence of population limitation and cytokine stimulus | 0.0174 | h^-1^ | [^132^](#_ENREF_132) | Cell cycle ~40 hours |
| *r*_microglia_ | Average basal proliferation rate of microglia obtained in the absence of population limitation and cytokine stimulus | 0.0174 | h^-1^ | [^133-135^](#_ENREF_133) | Cell cycle ~40 hours |
| *r*_astrocyte_ | Average basal proliferation rate of astrocyte obtained in the absence of population limitation and cytokine stimulus | 0.0174 | h^-1^ | [^130^](#_ENREF_130) | Cell cycle ~40 hours |
| *x*_max_ | Maximum cell population (including QSC, ASC, glioma, microglia, astrocyte) level due to limited nutrition and space | 3E7 | ml^-1^ |  | Density-dependent inhibition of cell growth |
| *u*cell_IL1 | Maximum up-regulation ratio of maximum cell concentration due to angiogenic effect of IL1 | 0.1 |  |  |  |
| *u*cell_VEGF | Maximum up-regulation ratio of maximum cell concentration due to angiogenic effect of VEGF | 0.2 |  |  |  |
| *u*cell_FGF | Maximum up-regulation ratio of maximum cell concentration due to angiogenic effect of FGF | 0.2 |  |  |  |
| *u*cell_HGF | Maximum up-regulation ratio of maximum cell concentration due to angiogenic effect of HGF | 0.1 |  |  |  |
| *u*cell_MIF | Maximum up-regulation ratio of maximum cell concentration due to angiogenic effect of MIF | 0.1 |  |  |  |
| *u*cell_SCF | Maximum up-regulation ratio of maximum cell concentration due to angiogenic effect of SCF | 0.15 |  | [^21^](#_ENREF_21) |  |
| *u*QSC_FGF | Maximum up-regulation ratio of QSC activation rate by FGF | 2 |  | [^1^](#_ENREF_1) |  |
| *u*ASC_EGF | Maximum up-regulation ratio of ASC proliferation rate by EGF | 2 |  | [^2^](#_ENREF_2) |  |
| *u*glio_ASC_IL6 | Maximum up-regulation ratio of differentiation rate from ASC to glioma by IL6 | 2 |  | [^4^](#_ENREF_4) |  |
| *u*glio_IL1 | Maximum up-regulation ratio of glioma proliferation rate by IL1 | 2 |  |  |  |
| *u*glio_IL6 | Maximum up-regulation ratio of glioma proliferation rate by IL6 | 2 |  | [^29^](#_ENREF_29) |  |
| *u*glio_IL10 | Maximum up-regulation ratio of glioma proliferation rate by IL10 | 2 |  | [^33^](#_ENREF_33) |  |
| *u*glio_TGFβ | Maximum up-regulation ratio of glioma proliferation rate by TGFβ | 2 |  |  |  |
| *u*glio_EGF | Maximum up-regulation ratio of glioma proliferation rate by EGF | 3 |  |  |  |
| *u*glio_VEGF | Maximum up-regulation ratio of glioma proliferation rate by VEGF | 3 |  | [^42^](#_ENREF_42) |  |
| *u*glio_HGF | Maximum up-regulation ratio of glioma proliferation rate by HGF | 3 |  | [^46^](#_ENREF_46) |  |
| *u*glio_GCSF | Maximum up-regulation ratio of glioma proliferation rate by GCSF | 2 |  | [^49^](#_ENREF_49) |  |
| *u*glio_SCF | Maximum up-regulation ratio of glioma proliferation rate by SCF | 2 |  |  |  |
| *u*glio_MIF | Maximum up-regulation ratio of glioma proliferation rate by MIF | 2 |  |  |  |
| *u*MIF_glio | Maximum up-regulation ratio of secretion rate of MIF from glioma due to hypoxia and hypoglycemia | 6 |  | [^10^](#_ENREF_10) |  |
| *u*micro_MCP1 | Maximum up-regulation ratio of replenishment rate of microglia due to chemotaxis | 5 |  |  |  |
| *u*micro_EGF | Maximum up-regulation ratio of microglia proliferation rate by EGF | 2 |  | [^63^](#_ENREF_63) |  |
| *u*micro_VEGF | Maximum up-regulation ratio of microglia proliferation rate by VEGF | 2 |  | [^64^](#_ENREF_64) |  |
| *u*micro_HGF | Maximum up-regulation ratio of microglia proliferation rate by HGF | 2 |  |  |  |
| *u*micro_GMCSF | Maximum up-regulation ratio of microglia proliferation rate by GMCSF | 2 |  |  |  |
| *u*astro_IL1 | Maximum up-regulation ratio of astrocyte proliferation rate by IL-1 | 2.5694 |  |  |  |
| *u*astro_PGE2 | Maximum up-regulation ratio of astrocyte proliferation rate by PGE2 | 2 |  | [^75^](#_ENREF_75) |  |
| *u*IL6_IL1 | Maximum up-regulation ratio of IL-6 production by glioma in the presence of IL-1β | 2 |  | [^83^](#_ENREF_83) |  |
| *u*TGFβ_IL1 | Maximum up-regulation ratio of TGF-β production by glioma in the presence of IL-1β | 2 |  |  |  |
| *u*GMCSF_IL10 | Maximum up-regulation ratio of decay rate of GM-CSF in the presence of IL-10 | 1 |  | [^115-117^](#_ENREF_115) |  |
| *u*GCSF_IL10 | Maximum up-regulation ratio of decay rate of G-CSF in the presence of IL-10 | 1 |  |  |  |
| *u*VEGF_TNFα | Maximum up-regulation ratio of VEGF production by glioma in the presence of TNFα | 2 |  | [^43^](#_ENREF_43) |  |
| *u*MIF_TNFα | Maximum up-regulation ratio of MIF production by microglia in the presence of TNFα | 2 |  |  |  |
| *u*VEGF_MIF | Maximum up-regulation ratio of VEGF production by glioma in the presence of MIF | 2 |  |  |  |
| *u*HGF_PGE2 | Maximum up-regulation ratio of HGF production by microglia in the presence of PGE2 | 2 |  | [^65^](#_ENREF_65) |  |
| *s*_glioma_ | Half max concentration of glioma cell in hypoxia dependent MIF production | 1.5e7 | ml^-1^ | [^10^](#_ENREF_10) |  |
| *s*_IL1_ | Saturation concentration of IL-1 producing half max regulation effect to cellular activity | 2200 | pM | Estimated |  |
| *s*_IL6_ | Saturation concentration of IL-6 producing half max regulation effect to cellular activity | 2200 | pM | Estimated |  |
| *s*_IL10_ | Saturation concentration of IL-10 producing half max regulation effect to cellular activity | 2200 | pM | Estimated |  |
| *s*_TGFβ_ | Saturation concentration of TGFβ producing half max regulation effect to cellular activity | 2200 | pM | Estimated |  |
| *s*_TNFα_ | Saturation concentration of TNFα producing half max regulation effect to cellular activity | 2200 | pM | Estimated |  |
| *s*_EGF_ | Saturation concentration of EGF producing half max regulation effect to cellular activity | 2200 | pM | Estimated |  |
| *s*_VEGF_ | Saturation concentration of VEGF producing half max regulation effect to cellular activity | 2200 | pM | Estimated |  |
| *s*_FGF_ | Saturation concentration of FGF producing half max regulation effect to cellular activity | 2200 | pM | Estimated |  |
| *s*_HGF_ | Saturation concentration of HGF producing half max regulation effect to cellular activity | 2200 | pM | Estimated |  |
| *s*_GCSF_ | Saturation concentration of G-CSF producing half max regulation effect to cellular activity | 2200 | pM | Estimated |  |
| *s*_SCF_ | Saturation concentration of SCF producing half max regulation effect to cellular activity | 2200 | pM | Estimated |  |
| *s*_PGE2_ | Saturation concentration of PGE2 producing half max regulation effect to cellular activity | 2200 | pM | Estimated |  |
| *s*_MCP1_ | Saturation concentration of MCP1 producing half max regulation effect to cellular activity | 1100 | pM | Estimated |  |
| *s*_GMCSF_ | Saturation concentration of GM-CSF producing half max regulation effect to cellular activity | 2200 | pM | Estimated |  |
| *s*_MIF_ | Saturation concentration of MIF producing half max regulation effect to cellular activity | 2200 | pM | Estimated |  |
| *d*_QSC_ | Death rate of quiescent glioma stem cell based on lifespan | 0.00001 | h^-1^ | [^144-146^](#_ENREF_144) | $\frac{\ln2}{\text{cell} \text{half-life}}$ Stem cell half-life ~8 years |
| *d*_ASC_ | Death rate of activated glioma stem cell based on lifespan | 0.0001 | h^-1^ | [^144-146^](#_ENREF_144) | Half-life ~9.6 months |
| *d*_glioma_ | Death rate of glioma cell based on lifespan | 0.001 | h^-1^ | [^147^](#_ENREF_147) | Half-life ~29 days |
| *d*_microglia_ | Death rate of microglia based on lifespan | 0.001 | h^-1^ | [^147^](#_ENREF_147) | Half-life ~29 days |
| *d*_astrocyte_ | Death rate of astrocyte based on lifespan | 0.001 | h^-1^ | [^147^](#_ENREF_147) | Half-life ~29 days |
| *d*_IL1_ | Decay rate of IL-1 based on half-life | 0.6931 | h^-1^ | [^148-153^](#_ENREF_148) | Half-life ~1 hour |
| *d*_IL6_ | Decay rate of IL-6 based on half-life | 0.6931 | h^-1^ | [^148-153^](#_ENREF_148) | Half-life ~1 hour |
| *d*_IL10_ | Decay rate of IL-10 based on half-life | 0.6931 | h^-1^ | [^148-153^](#_ENREF_148) | Half-life ~1 hour |
| *d*_TNFα_ | Decay rate of TNFα based on half-life | 0.6931 | h^-1^ | [^148-153^](#_ENREF_148) | Half-life ~1 hour |
| *d*_TGFβ_ | Decay rate of TGFβ based on half-life | 0.6931 | h^-1^ | [^148-153^](#_ENREF_148) | Half-life ~1 hour |
| *d*_EGF_ | Decay rate of EGF based on half-life | 0.6931 | h^-1^ | [^148-153^](#_ENREF_148) | Half-life ~1 hour |
| *d*_VEGF_ | Decay rate of VEGF based on half-life | 0.6931 | h^-1^ | [^148-153^](#_ENREF_148) | Half-life ~1 hour |
| *d*_FGF_ | Decay rate of FGF based on half-life | 0.6931 | h^-1^ | [^148-153^](#_ENREF_148) | Half-life ~1 hour |
| *d*_HGF_ | Decay rate of HGF based on half-life | 0.6931 | h^-1^ | [^148-153^](#_ENREF_148) | Half-life ~1 hour |
| *d*_MCP1_ | Decay rate of MCP1 based on half-life | 0.6931 | h^-1^ | [^148-153^](#_ENREF_148) | Half-life ~1 hour |
| *d*_MIF_ | Decay rate of MIF based on half-life | 0.6931 | h^-1^ | [^148-153^](#_ENREF_148) | Half-life ~1 hour |
| *d*_PGE2_ | Decay rate of PGE2 based on half-life | 0.6931 | h^-1^ | [^148-153^](#_ENREF_148) | Half-life ~1 hour |
| *d*_GMCSF_ | Decay rate of GM-CSF based on half-life | 0.6931 | h^-1^ | [^148-153^](#_ENREF_148) | Half-life ~1 hour |
| *d*_GCSF_ | Decay rate of G-CSF based on half-life | 0.6931 | h^-1^ | [^148-153^](#_ENREF_148) | Half-life ~1 hour |
| *d*_SCF_ | Decay rate of SCF based on half-life | 0.6931 | h^-1^ | [^148-153^](#_ENREF_148) | Half-life ~1 hour |
| *k*QSC_ASC | Deactivation rate | 1e-3 | h^-1^ | Estimated |  |
| *k*ASC_QSC | Activation rate | 5e-3 | h^-1^ | Estimated |  |
| *p*ASC_glio | Probability of dividing glioma dedifferentiate to ASC during each cell cycle | 1e-4 |  | Estimated |  |
| *p*glio_ASC | Probability of dividing ASC differentiate to glioma during each cell cycle | 0.5 |  | [^161^](#_ENREF_161) |  |
| *p*glio_astro | Probability of dividing astrocyte mutate to glioma during each cell cycle | 1E-6 |  | [^162-164^](#_ENREF_162) |  |
| *k*IL1_glio | Secretion rate of IL1 by glioma | 60 | 10^-21^mol h^-1^ | [^80-82^](#_ENREF_80) |  |
| *k*IL1_micro | Secretion rate of IL1 by microglia | 22.1 | 10^-21^mol h^-1^ |  |  |
| *k*IL1_astro | Secretion rate of IL1 by astrocyte | 3 | 10^-21^mol h^-1^ | [^76^](#_ENREF_76) |  |
| *k*IL6_glio | Secretion rate of IL6 by glioma | 40 | 10^-21^mol h^-1^ |  |  |
| *k*IL6_micro | Secretion rate of IL6 by microglia | 96.2 | 10^-21^mol h^-1^ |  |  |
| *k*IL6_astro | Secretion rate of IL6 by astrocyte | 6 | 10^-21^mol h^-1^ |  |  |
| *k*IL10_micro | Secretion rate of IL10 by microglia | 60 | 10^-21^mol h^-1^ |  |  |
| *k*TNFα_micro | Secretion rate of TNFα by microglia | 29.4 | 10^-21^mol h^-1^ |  |  |
| *k*TNFα_astro | Secretion rate of TNFα by astrocyte | 2.94 | 10^-21^mol h^-1^ |  |  |
| *k*TGFβ_ASC | Secretion rate of TGFβ by ASC | 0.15 | 10^-21^mol h^-1^ | [^97^](#_ENREF_97) |  |
| *k*TGFβ_glio | Secretion rate of TGFβ by glioma | 40 | 10^-21^mol h^-1^ | [^31^](#_ENREF_31) |  |
| *k*TGFβ_micro | Secretion rate of TGFβ by microglia | 60 | 10^-21^mol h^-1^ | [^100-102^](#_ENREF_100) |  |
| *k*EGF_glio | Secretion rate of EGF by glioma | 60 | 10^-21^mol h^-1^ | [^104^](#_ENREF_104) |  |
| *k*EGF_micro | Secretion rate of EGF by microglia | 60 | 10^-21^mol h^-1^ | [^103^](#_ENREF_103) |  |
| *k*VEGF_ASC | Secretion rate of VEGF by ASC | 30 | 10^-21^mol h^-1^ |  |  |
| *k*VEGF_glio | Secretion rate of VEGF by glioma | 60 | 10^-21^mol h^-1^ |  |  |
| *k*VEGF_micro | Secretion rate of VEGF by microglia | 60 | 10^-21^mol h^-1^ | [^13^](#_ENREF_13) |  |
| *k*FGF_ASC | Secretion rate of FGF by ASC | 60 | 10^-21^mol h^-1^ |  |  |
| *k*FGF_glio | Secretion rate of FGF by glioma | 60 | 10^-21^mol h^-1^ | [^5^](#_ENREF_5) |  |
| *k*HGF_glio | Secretion rate of HGF by glioma | 60 | 10^-21^mol h^-1^ |  |  |
| *k*HGF_micro | Secretion rate of HGF by microglia | 60 | 10^-21^mol h^-1^ | [^65^](#_ENREF_65) |  |
| *k*MCP1_glio | Secretion rate of MCP1 by glioma | 90 | 10^-21^mol h^-1^ | [^58-60^](#_ENREF_58) |  |
| *k*MIF_glio | Secretion rate of MIF by glioma | 20 | 10^-21^mol h^-1^ | [^110^](#_ENREF_110) |  |
| *k*MIF_micro | Secretion rate of MIF by microglia | 15 | 10^-21^mol h^-1^ | [^20^](#_ENREF_20) |  |
| *k*PGE2_micro | Secretion rate of PGE2 by microglia | 60 | 10^-21^mol h^-1^ |  |  |
| *k*GMCSF_glio | Secretion rate of GM-CSF by glioma | 60 | 10^-21^mol h^-1^ |  |  |
| *k*GMCSF_micro | Secretion rate of GM-CSF by microglia | 60 | 10^-21^mol h^-1^ |  |  |
| *k*GCSF_glio | Secretion rate of G-CSF by glioma | 60 | 10^-21^mol h^-1^ |  |  |
| *k*SCF_glio | Secretion rate of SCF by glioma | 60 | 10^-21^mol h^-1^ |  |  |
| *k*SCF_micro | Secretion rate of SCF by microglia | 60 | 10^-21^mol h^-1^ |  |  |
