# Supplementary material for: In silico Experimentation of Glioma Microenvironment Development and Anti-tumor Therapy
Source: PLoS Comput Biol. 2012 Feb 2;8(2):e1002355. doi: 10.1371/journal.pcbi.1002355 (PMC3271023; doi:10.1371/journal.pcbi.1002355)
Supplement: Table S2 — Stochastic parameters. (DOCX) [file pcbi.1002355.s008.docx]

**Supplementary Table S2. Stochastic Parameters**

| Deterministic variable | Stochastic description | Parameters | Dimension | Comment |
| --- | --- | --- | --- | --- |
| *r*_ASC_ | *r*(1+*ε*sin(Ω*t*+*σW*(*t*)+Δ))  *r*_ASC_=0.0005  *ε*_ASC_=0.99  Ω_ASC_=0.0045  *σ*_ASC_=10 | *r*_ASC_ is the basal proliferation rate of ASC. *ε* is the amplitude of stochastic fluctuation; *σ* is the bandwidth factor ; Δ is a random phase uniformly distributed in [0, 2π]. | Ω (h^-1^),  Others are dimensionless | *r* > 0,  0 ≤ *ε*< 1, $\Omega=\frac{2\pi r_{\text{ASC}}}{\ln2}$  |
| *r*_glioma_ | *r*(1+*ε*sin(Ω*t*+*σW*(*t*)+Δ))  *r*_glioma_=0.0174  *ε*_glioma_=0.99  Ω_glioma_=0.1577  *σ*_glioma_=10 | *r*_glioma_ is the basal proliferation rate of glioma. | Ω (h^-1^),  Others are dimensionless | *r* > 0,  0 ≤ *ε*< 1, $\Omega=\frac{2\pi r_{\text{glioma}}}{\ln2}$  |
| *r*_astrocyte_ | *r*(1+*ε*sin(Ω*t*+*σW*(*t*)+Δ))  *r*_astrocyte_=0.0174  *ε*_astrocyte_=0.99  Ω_astrocyte_=0.1577  *σ*_astrocyte_=10 | *r*_astrocyte_ is the basal proliferation rate of astrocyte. | Ω (h^-1^),  Others are dimensionless | *r*>0,  0 ≤ *ε*< 1, $\Omega=\frac{2\pi r_{\text{astroglia}}}{\ln2}$  |
| *r*_microglia_ | *r*(1+*ε*sin(Ω*t*+*σW*(*t*)+Δ))  *r*_microglia_=0.0174  *ε*_microglia_=0.99  Ω_microglia_=0.1577  *σ*_microglia_=10 | *r*_microglia_ is the basal proliferation rate of microglia. | Ω (h^-1^),  Others are dimensionless | *r*>0,  0 ≤ *ε*< 1, $\Omega=\frac{2\pi r_{\text{microglia}}}{\ln2}$  |
| *p*_glio_astro_ | *p*(1+*ε*sin(Ω*t*+*σW*(*t*)+Δ));  *p*_glio_astro_=1×10^-6^  *ε*_glio_astro_=1  Ω_glio_astro_=0.1577  *σ*_glio_astro_=10 | *p*_glio_astro_ is the average proportion of dividing astroglia mutate to glioma during each cell cycle; | Ω (h^-1^),  Others are dimensionless | $0\leq p\leq1$, $0\leq\varepsilon\leq\min\left(1,\frac{1}{p}-1\right)$ $\Omega=\frac{2\pi}{T_{\text{cell} \text{cycle}}}=\frac{2\pi r_{\text{astroglia}}}{\ln2}$    |
| *p*_glio_ASC_ | *p*(1+*ε*sin(Ω*t*+*σW*(*t*)+Δ));  *p*_glio_ASC_=0.5  *ε*_glio_ASC_=1  Ω_glio_ASC_=0.0045  *σ*_glio_ASC_=10 | *p*_glio_ASC_ is the average proportion of dividing ASC differentiate to glioma during each cell cycle. | Ω (h^-1^),  Others are dimensionless | $0\leq p\leq1$, $0\leq\varepsilon\leq\min\left(1,\frac{1}{p}-1\right)$ $\Omega=\frac{2\pi}{T_{\text{cell} \text{cycle}}}=\frac{2\pi r_{\text{ASC}}}{\ln2}$    |
| *p*_ASC_glio_ | *p*(1+*ε*sin(Ω*t*+*σW*(*t*)+Δ));  *p*_ASC_glio_=1×10^-4^  *ε*_ASC_glio_=1  Ω_ASC_glio_=0.1577  *σ*_ASC_glio_=10 | *p*_ASC_glio_ is the average proportion of dividing glioma dedifferentiate to ASC during each cell cycle. | Ω (h^-1^),  Others are dimensionless | $0\leq p\leq1$, $0\leq\varepsilon\leq\min\left(1,\frac{1}{p}-1\right)$ $\Omega=\frac{2\pi}{T_{\text{cell} \text{cycle}}}=\frac{2\pi r_{\text{glioma}}}{\ln2}$    |
| *c*_QSC_ | $\xi(t)=\sum_{k=1}^{N(t)} Y_{k}\delta(t-\tau_{k})$;  *Y*_k_=1;  *λ*=*c*_QSC_=1; | *c*_QSC_ is the average rate of supply of quiescent glioma stem cell from normal neural stem cell;  *ξ*(*t*) is Poisson white noise;  *N*(*t*) is Poisson counting process giving the number of pulses that arrive in the time interval [0, *t*];  *λ* is the mean arrival rate of *N*(*t*); |  |  |
| *c*_microglia_ | $\xi(t)=\sum_{k=1}^{N(t)} Y_{k}\delta(t-\tau_{k})$;  *Y_k_*=1;  *λ*=$c_{microglia}\left(1+\frac{u_{micro\_MCP1}y_{MCP1}}{s_{MCP1}+y_{MCP1}}\right)$; | *c*_microglia_ is the average rate of supply of microglia from monocytes; |  |  |
| *c*_astroglia_ | $\xi(t)=\sum_{k=1}^{N(t)} Y_{k}\delta(t-\tau_{k})$;  *Y*_k_=1;  *λ*=*c*_astroglia_; | *c*_astroglia_ is the average rate of supply of astroglia from progenitors; |  |  |
| *k_i_* | *k_i_*max(0,1+*σN*(0,1)) | Truncated normal distribution |  |  |
