# Supplementary material for: In silico Experimentation of Glioma Microenvironment Development and Anti-tumor Therapy
Source: PLoS Comput Biol. 2012 Feb 2;8(2):e1002355. doi: 10.1371/journal.pcbi.1002355 (PMC3271023; doi:10.1371/journal.pcbi.1002355)
Supplement: Table S3 — Patients parameters for Figure S5(a). (DOCX) [file pcbi.1002355.s009.docx]

**Supplementary Table S3. Four patients with difference in six regulation ratio parameters (Patients parameters for Supplementary Figure S5(a))**

|  | Patient #1 | Patient #2 | Patient #3 | Patient #4 |
| --- | --- | --- | --- | --- |
| *u*_glio_IL6_ | 0.5 | 5 | 0.5 | 5 |
| *u*_glio_EGF_ | 0.5 | 0.5 | 5 | 0.5 |
| *u*_glio_VEGF_ | 5 | 0.5 | 5 | 5 |
| *u*_glio_HGF_ | 6 | 0.3 | 0.3 | 0.3 |
| *u*_astro_PGE2_ | 1 | 3 | 1 | 1 |
| *u*_astro_IL1_ | 1 | 1 | 0.2 | 1 |
