# Supplementary material for: In silico Experimentation of Glioma Microenvironment Development and Anti-tumor Therapy
Source: PLoS Comput Biol. 2012 Feb 2;8(2):e1002355. doi: 10.1371/journal.pcbi.1002355 (PMC3271023; doi:10.1371/journal.pcbi.1002355)
Supplement: Table S4 — Patients parameters for Figure S5(b). (DOCX) [file pcbi.1002355.s010.docx]

**Supplementary Table S4. Four patients with difference in six secretion rate parameters (Patients parameters for Supplementary Figure S5(b))**

**(Unit: 10^-21^mol h^-1^)**

|  | Patient #1 | Patient #2 | Patient #3 | Patient #4 |
| --- | --- | --- | --- | --- |
| *k*_IL1_micro_ | 2 | 2 | 40 | 40 |
| *k*_IL1_astro_ | 0.3 | 0.3 | 6 | 6 |
| *k*_PGE2_micro_ | 6 | 120 | 6 | 120 |
| *k*_IL6_micro_ | 9 | 180 | 9 | 180 |
| *k*_HGF_micro_ | 6 | 120 | 120 | 6 |
| *k*_GMCSF_micro_ | 120 | 6 | 6 | 120 |
