# Supplementary material for: In silico Experimentation of Glioma Microenvironment Development and Anti-tumor Therapy
Source: PLoS Comput Biol. 2012 Feb 2;8(2):e1002355. doi: 10.1371/journal.pcbi.1002355 (PMC3271023; doi:10.1371/journal.pcbi.1002355)
Supplement: Table S5 — Parameter profiles of three virtual patients for Figure 3 and 4. (DOCX) [file pcbi.1002355.s011.docx]

**Supplementary Table S5. Three virtual patients with different cytokine secretion rates profiles**

**(Unit: 10^-21^mol h^-1^)**

|  | Patient #1 | Patient #2 | Patient #3 |
| --- | --- | --- | --- |
| *k*_IL1_glio_ | 60 | 20 | 15 |
| *k*_IL1_micro_ | 30 | 15 | 15 |
| *k*_IL1_astro_ | 5 | 3 | 1 |
| *k*_IL6_glio_ | 60 | 20 | 10 |
| *k*_IL6_micro_ | 100 | 50 | 20 |
| *k*_IL6_astro_ | 10 | 5 | 1 |
| *k*_IL10_micro_ | 10 | 40 | 90 |
| *k*_TNFα_micro_ | 10 | 20 | 40 |
| *k*_TNFα_astro_ | 1 | 2 | 4 |
| *k*_TGFβ_ASC_ | 0.15 | 0.15 | 0.15 |
| *k*_TGFβ_glio_ | 10 | 40 | 110 |
| *k*_TGFβ_micro_ | 15 | 50 | 90 |
| *k*_EGF_glio_ | 10 | 60 | 100 |
| *k*_EGF_micro_ | 10 | 60 | 100 |
| *k*_VEGF_ASC_ | 30 | 30 | 30 |
| *k*_VEGF_glio_ | 90 | 60 | 30 |
| *k*_VEGF_micro_ | 90 | 60 | 30 |
| *k*_FGF_ASC_ | 60 | 60 | 120 |
| *k*_FGF_glio_ | 60 | 60 | 120 |
| *k*_HGF_glio_ | 90 | 20 | 10 |
| *k*_HGF_micro_ | 90 | 60 | 40 |
| *k*_MCP1_glio_ | 90 | 90 | 90 |
| *k*_MIF_glio_ | 40 | 15 | 5 |
| *k*_MIF_micro_ | 30 | 10 | 5 |
| *k*_PGE2_micro_ | 10 | 30 | 40 |
| *k*_GMCSF_glio_ | 60 | 60 | 60 |
| *k*_GMCSF_micro_ | 60 | 60 | 60 |
| *k*_GCSF_glio_ | 10 | 50 | 100 |
| *k*_SCF_glio_ | 10 | 70 | 120 |
| *k*_SCF_micro_ | 10 | 70 | 100 |
