# Supplementary material for: In silico Experimentation of Glioma Microenvironment Development and Anti-tumor Therapy
Source: PLoS Comput Biol. 2012 Feb 2;8(2):e1002355. doi: 10.1371/journal.pcbi.1002355 (PMC3271023; doi:10.1371/journal.pcbi.1002355)
Supplement: Table S6 — The x-coordinate parameter panels for Figure S5(a). (DOCX) [file pcbi.1002355.s012.docx]

**Supplementary Table S6. x-axis parameter panels of Supplementary Fig. S5(a)**

| x-axis label | Corresponding parameters |
| --- | --- |
| 1 | *k*_IL1_glio_ |
| 2 | *k*_IL1_micro_ |
| 3 | *k*_IL1_astro_ |
| 4 | *k*_IL6_glio_ |
| 5 | *k*_IL6_micro_ |
| 6 | *k*_IL6_astro_ |
| 7 | *k*_IL10_micro_ |
| 8 | *k*_TNFα_micro_ |
| 9 | *k*_TNFα_astro_ |
| 10 | *k*_TGFβ_ASC_ |
| 11 | *k*_TGFβ_glio_ |
| 12 | *k*_TGFβ_micro_ |
| 13 | *k*_EGF_glio_ |
| 14 | *k*_EGF_micro_ |
| 15 | *k*_VEGF_ASC_ |
| 16 | *k*_VEGF_glio_ |
| 17 | *k*_VEGF_micro_ |
| 18 | *k*_FGF_ASC_ |
| 19 | *k*_FGF_glio_ |
| 20 | *k*_HGF_glio_ |
| 21 | *k*_HGF_micro_ |
| 22 | *k*_MCP1_glio_ |
| 23 | *k*_MIF_glio_ |
| 24 | *k*_MIF_micro_ |
| 25 | *k*_PGE2_micro_ |
| 26 | *k*_GMCSF_glio_ |
| 27 | *k*_GMCSF_micro_ |
| 28 | *k*_GCSF_glio_ |
| 29 | *k*_SCF_glio_ |
| 30 | *k*_SCF_micro_ |
