# Supplementary material for: In silico Experimentation of Glioma Microenvironment Development and Anti-tumor Therapy
Source: PLoS Comput Biol. 2012 Feb 2;8(2):e1002355. doi: 10.1371/journal.pcbi.1002355 (PMC3271023; doi:10.1371/journal.pcbi.1002355)
Supplement: Table S7 — The x-coordinate parameter panels for Figure 4(a) and Figure S5(b). (DOCX) [file pcbi.1002355.s013.docx]

**Supplementary Table S7. x-axis parameter panels of Fig. 4(a) and Supplementary Fig. S5(b)**

| x-axis label | Corresponding parameters |
| --- | --- |
| 1 | *u*_QSC_FGF_ |
| 2 | *u*_ASC_EGF_ |
| 3 | *u*_glio_ASC_IL6_ |
| 4 | *u*_glio_IL1_ |
| 5 | *u*_glio_IL6_ |
| 6 | *u*_glio_IL10_ |
| 7 | *u*_glio_TGFβ_ |
| 8 | *u*_glio_EGF_ |
| 9 | *u*_glio_VEGF_ |
| 10 | *u*_glio_HGF_ |
| 11 | *u*_glio_GCSF_ |
| 12 | *u*_glio_SCF_ |
| 13 | *u*_glio_MIF_ |
| 14 | *u*_MIF_glio_ |
| 15 | *u*_micro_MCP1_ |
| 16 | *u*_micro_EGF_ |
| 17 | *u*_micro_VEGF_ |
| 18 | *u*_micro_HGF_ |
| 19 | *u*_micro_GMCSF_ |
| 20 | *u*_astro_IL1_ |
| 21 | *u*_astro_PGE2_ |
| 22 | *u*_IL6_IL1_ |
| 23 | *u*_TGFβ_IL1_ |
| 24 | *u*_GMCSF_IL10_ |
| 25 | *u*_GCSF_IL10_ |
| 26 | *u*_VEGF_TNFα_ |
| 27 | *u*_MIF_TNFα_ |
| 28 | *u*_VEGF_MIF_ |
| 29 | *u*_HGF_PGE2_ |
