# Supplementary material for: In silico Experimentation of Glioma Microenvironment Development and Anti-tumor Therapy
Source: PLoS Comput Biol. 2012 Feb 2;8(2):e1002355. doi: 10.1371/journal.pcbi.1002355 (PMC3271023; doi:10.1371/journal.pcbi.1002355)
Supplement: Text S1 — Section 1: Deterministic description of the intercellular signaling network. Section 2: Stochastic description of rate parameters. Section 3: Sensitivity analysis. (PDF) [file pcbi.1002355.s014.pdf]

Supporting Text

I. Supporting Figure Legends

Supporting Figure S1 The growth rare of glioma tumor is dictated by ASC differentiation and astrocyte mutation to glioma cells. The contributions of these two factors are comparable in the first pre-cancer phase. However, neoplastic transformation of astrocytes directly to glioma cells contributes little to tumor development in the rapid expansion and malignant phases.

Supporting Figure S2 Influence of initial conditions to tumorigenesis time is demonstrated by perturbing the initial concentration of QSC and microglia. (**a**) Three different initial concentration of QSC are compared. With a higher initial QSC concentration, the patient will get faster progression. However, the influence is not significant. (**b**) The initial concentration of microglia has a striking effect on tumorigenesis time.

Supporting Figure S3 Virtual therapy of patient #3 demonstrates the difference of therapeutic efficacy between single-targeted and combination-targeted. The monotherapy targeted at even the most significant signaling cannot arrest the rapid progression, while the combinational therapy leads to a significant synergistic effect on tumor suppression.

Supporting Figure S4 Virtual therapies of two patients demonstrate the therapeutic efficacy of combination-targeted therapy. Concomitant improvement of patient survival is observed during expansion of targeted cytokine. (**a**) Virtual treatment for patient #1, up to four cytokine combination is adopted. (**b**) Treatment for patient #3. The expected therapeutic effect is achieved by targeting five signaling simultaneously.

Supporting Figure S5 Three patients are treated with the same protocol, which is personalized according to the cytokine secretion profile of patient #3. Patient #3 receives expected therapeutic effect, while the progression of patient #2 also slows down. However, the failure of patient #1 suggests missing the target. (**a**) Patient #1. (**b**) Patient #2. (**c**) Patient #3.

Supporting Figure S6 Inter-patient heterogeneity was demonstrated by sensitivity analyses of signaling-related parameters for tumorigenesis time. a. Sensitivity factors of cytokine secretion rates. Four patients with difference in six parameters (Supporting Table S3) are compared. The x-axis parameter panels are listed in Supporting Table S6. b. Sensitivity factors of receptor signaling regulation ratios. Twenty nine parameters are analyzed and four patients with difference in six parameters (Supporting Table S4) are compared. The x-coordinate parameter panels are listed in Supporting Table S7.

II. Supporting Methods

1. Deterministic description of the intercellular signaling network.

We translated the intercellular signaling network Fig. 1a into mathematical format by employing population dynamics and introducing stochastic mechanism. The deterministic descriptions are dozens of ordinary differential equations as follows. The meanings of parameters are listed in Supporting Table S1. The computational codes are available upon request, which should be addressed to R.F. or Y.W.

Quiescent glioma stem cell (QSC)

FGF (FGF7 and FGF10) signaling contributes to the telogen to anagen transition, adding new insights into the process of stem cell activation[1].

$$\dot{x}_{QSC} = c_{QSC} + K_{QSC\_ASC} - K_{ASC\_QSC} - d_{QSC}x_{QSC} \tag{1}$$

where

$$K_{QSC\_ASC} = k_{QSC\_ASC}x_{ASC}$$

$$K_{ASC\_QSC} = k_{ASC\_QSC}\left(1 + \frac{u_{QSC\_FGF}y_{FGF}}{s_{FGF} + y_{FGF}}\right)x_{QSC}$$

K_{QSC_ASC} is the increase due to deactivation/quiescence of ASC, and K_{ASC_QSC} describes the decrease because of activation of QSC.

Activated glioma stem cell (ASC)

EGF and FGF2 enhanced GBM brain tumor stem cells survival, proliferation, and subsequent sphere size [2].

Virtually all neural stem cells maintain an undifferentiated state and the capacity to self-renew in response to FGF2 [3].

IL6 signaling contributes to glioma malignancy through the promotion of GSC growth and survival [4].

Up-regulation of FGF5 during malignant progression might reflect dedifferentiation and acquisition of stem cell-like properties [5].

VEGF, FGF, SCF, IL1, HGF, and MIF are recognized as major factors that induce angiogenesis within GBM [6-21].

$$\dot{x}_{ASC} = K_{ASC\_QSC} - K_{QSC\_ASC} + R_{glioma} p_{ASC\_glio}\left(\frac{y_{FGF}}{s_{FGF} + y_{FGF}}\right) + R_{ASC}\left(1 - p_{glio\_ASC}\left(\frac{s_{FGF}}{s_{FGF} + y_{FGF}}\right)\left(\frac{u_{glio\_ASC\_IL6} y_{IL6}}{s_{IL6} + y_{IL6}}\right)\right) - d_{ASC} x_{ASC} \quad (2)$$

where

$$R_{ASC} = r_{ASC} x_{ASC}\left(1 - \frac{x_{QSC} + x_{ASC} + x_{glioma} + x_{astroglia} + x_{microglia}}{x_{\max} A_{angiogenesis}}\right)\left(1 + \frac{u_{ASC\_EGF} y_{EGF}}{s_{EGF} + y_{EGF}}\right)$$

$$A_{angiogenesis} = \left(1 + \frac{u_{cell\_IL1} y_{IL1}}{s_{IL1} + y_{IL1}}\right)\left(1 + \frac{u_{cell\_VEGF} y_{VEGF}}{s_{VEGF} + y_{VEGF}}\right)\left(1 + \frac{u_{cell\_HGF} y_{HGF}}{s_{HGF} + y_{HGF}}\right)\left(1 + \frac{u_{cell\_MIF} y_{MIF}}{s_{MIF} + y_{MIF}}\right)\left(1 + \frac{u_{cell\_SCF} y_{SCF}}{s_{SCF} + y_{SCF}}\right)\left(1 + \frac{u_{cell\_FGF} y_{FGF}}{s_{FGF} + y_{FGF}}\right)$$

R_{ASC} is the logistic proliferation term. Parameter x_{max} is the saturating concentration factor, whereas $A_{angiogenesis}$ is the angiogenesis factor. So the product $x_{max}A_{angiogenesis}$ represents the carrying capacity. The first term of the right-hand side of Eq. (2) is the activation term, and the second term is the deactivation term. The third term is the dedifferentiation from glioma cells to ASC, while the fourth term is the result of proliferation minus differentiation. The last term is the decay of ASC.

Glioma

The experimental glioma genesis models indicate that when sufficient numbers of critical pathways are disrupted, glioma can originate from cells at all differentiation stages during glial cell development. In addition, progenitor cells appear to be more susceptible to transformation compared to the mature glial cells [22,23].

The Fibroblast Growth Factor (FGF) signaling pathway is reported to stimulate glioblastoma (GBM) growth [24,25]. Autocrine FGF5 is predominantly a survival and migration factor for GBM cells [5].

EGF receptor signalling promotes proliferation, tissue invasion, increases chemoresistance and inhibits apoptosis of glioma cells [26-28].

IL-1, IL-6, IL-10, TGFβ and their receptors were strongly expressed in nearly all glioblastomas and cell lines tested, and have been postulated to promote glioma cell proliferation [29,30,31-33].

The overexpression of EGF receptors suggests the potential for autocrine/paracrine proliferation in response to EGF and Hb-EGF [34-40].

TNF-α increases EGF receptor expression in glioma cells in vitro [41].

A decrease in tumor-cell proliferation was observed in *vivo* by systemic treatment with a monoclonal antibody against VEGFR-2 [42].

TNF-α increases VEGF expression in glioma cells in vitro [43].

HGF and its receptor, Met, have been found in gliomas [44], where they are thought to be involved in cell motility, chemoattraction, and tumor invasion [45,46].

G-CSF/G-CSFR is expressed constitutively in some glioma cell lines [47,48] and in human gliomas, where it has been postulated to promote in an autocrine fashion glioma cell proliferation [49].

SCF and its receptor c-kit, are highly expressed in glioma cell lines [50,51], and SCF can mediate the proliferation of glioma cells in vitro [52].

MIF plays a particularly critical part in cell cycle regulation and therefore in tumorigenesis as well. [10,20,53,54]. Recent studies have suggested a potentially broader role for MIF in growth regulation because of its ability to antagonize p53-mediated gene activation and apoptosis [55].

PGE2 has been shown to transiently prevent glioma cell proliferation in vitro [56].

$$
\begin{aligned}
\dot{x}_{glioma} = {} & R_{ASC} p_{glio\_ASC} \left(\frac{s_{FGF}}{s_{FGF}+y_{FGF}}\right)\left(\frac{u_{glio\_ASC\_IL6}\, y_{IL6}}{s_{IL6}+y_{IL6}}\right) + p_{glio\_astro} R_{astroglia} \\
& + R_{glioma}\left(1 - p_{ASC\_glio}\left(\frac{y_{FGF}}{s_{FGF}+y_{FGF}}\right)\right) - d_{glioma} \frac{s_{EGF}}{s_{EGF}+y_{EGF}} \frac{s_{FGF}}{s_{FGF}+y_{FGF}} \frac{s_{MIF}}{s_{MIF}+y_{MIF}} x_{glioma}
\end{aligned} \tag{3}
$$

where

$$
\begin{aligned}
R_{glioma} = {} & r_{glioma} x_{glioma} \left(1 - \frac{x_{QSC}+x_{ASC}+x_{glioma}+x_{astroglia}+x_{microglia}}{x_{\max} A_{angiogenesis}}\right) \\
& \left(1+\frac{u_{glio\_IL1}\, y_{IL1}}{s_{IL1}+y_{IL1}}\right)\left(1+\frac{u_{glio\_IL6}\, y_{IL6}}{s_{IL6}+y_{IL6}}\right)\left(1+\frac{u_{glio\_IL10}\, y_{IL10}}{s_{IL10}+y_{IL10}}\right)\left(1+\frac{u_{glio\_TGF\beta}\, y_{TGF\beta}}{s_{TGF\beta}+y_{TGF\beta}}\right) \\
& \left(1+\frac{u_{glio\_EGF}\left(1+\frac{y_{TNF\alpha}}{s_{TNF\alpha}+y_{TNF\alpha}}\right) y_{EGF}}{s_{EGF}+y_{EGF}}\right)\left(1+\frac{u_{glio\_VEGF}\left(1+\frac{y_{TNF\alpha}}{s_{TNF\alpha}+y_{TNF\alpha}}\right) y_{VEGF}}{s_{VEGF}+y_{VEGF}}\right) \\
& \left(1+\frac{u_{glio\_HGF}\, y_{HGF}}{s_{HGF}+y_{HGF}}\right)\left(1+\frac{u_{glio\_GCSF}\, y_{GCSF}}{s_{GCSF}+y_{GCSF}}\right)\left(1+\frac{u_{glio\_SCF}\, y_{SCF}}{s_{SCF}+y_{SCF}}\right)\left(1+\frac{u_{glio\_MIF}\, y_{MIF}}{s_{MIF}+y_{MIF}}\right)\left(\frac{s_{PGE2}}{s_{PGE2}+y_{PGE2}}\right)
\end{aligned}
$$

R_{glioma} is the logistic proliferation term. The product $x_{max}A_{angiogenesis}$ indicates the carrying capacity. The first term of the right-hand side of Eq. (3) represents the differentiation from ASC to glioma cells. The second term describes the mutation from astrocytes to glioma cells. The third term is the result of proliferation minus dedifferentiation. The last term is the death of glioma cells due to life span.

Activated Microglia

It is generally accepted that monocytes are the most likely source of all brain macrophages. These cells, which begin their migration into normal brain during embryogenesis, can differentiate into microglia [57].

Glioma cells express the microglia chemoattractant, MCP-1, at the mRNA and protein levels [58-60], and microglia possess the specific MCP-1 receptor, CCR2 [61]. Thus, recruitment of microglia to the site of gliomas may in part result from the local production of MCP-1 [58,62].

Microglia express receptors for EGF that enable them to proliferate in response to local release of this growth factor [63].

In vitro, VEGF can also induce the proliferation and migration of microglia [64].

HGF and its receptor, c-Met, have been found in microglia [65], where they are thought to be microglial chemoattractant and inducer of proliferation in vitro [66].

GM-CSF is potent mitogen for microglia [67].

TGF-β inhibits the proliferation of microglia as well as their production of cytokines in vitro [68].

In vitro, SCF inhibits microglial proliferation and their expression of the inflammatory cytokines TNFa and IL-1b [69].

$$\dot{x}_{microglia} = c_{microglia}\left(1+\frac{u_{micro\_MCP1}y_{MCP1}}{s_{MCP1}+y_{MCP1}}\right)+r_{microglia}x_{microglia}\left(1-\frac{x_{QSC}+x_{ASC}+x_{glioma}+x_{astrocyte}+x_{microglia}}{x_{\max}A_{angiogenesis}}\right)$$
$$\left(1+\frac{u_{micro\_EGF}y_{EGF}}{s_{EGF}+y_{EGF}}\right)\left(1+\frac{u_{micro\_VEGF}y_{VEGF}}{s_{VEGF}+y_{VEGF}}\right)\left(1+\frac{u_{micro\_HGF}y_{HGF}}{s_{HGF}+y_{HGF}}\right) \quad (4)$$
$$\left(1+\frac{u_{micro\_GMCSF}y_{GMCSF}}{s_{GMCSF}+y_{GMCSF}}\right)\left(\frac{s_{TGF\beta}}{s_{TGF\beta}+y_{TGF\beta}}\right)\left(\frac{s_{SCF}}{s_{SCF}+y_{SCF}}\right)-d_{microglia}x_{microglia}$$

The first term of the right-hand side of Eq. (4) is the replenishment of microglia from monocytes. The second term is logistic proliferation.

Astrocyte

Astrocytes have been shown to originate from progenitors, and can migrate radially [70].

IL-1 has been shown to stimulate the growth of astrocytes in vitro [71-73]. The duration of survival of GBM patients is enhanced when levels of intratumoral IL-1β, not necessarily produced by microglia, are elevated [74].

PGE2 released from activated microglia enhances astrocyte proliferation in vitro [75].

$$\dot{x}_{astrocyte} = c_{astrocyte}+R_{astrocyte}(1-p_{glio\_astro})-d_{astrocyte}x_{astrocyte} \quad (5)$$

where

$$R_{astrocyte} = r_{astrocyte} x_{astrocyte} \left(1 - \frac{x_{QSC} + x_{ASC} + x_{glioma} + x_{astrocyte} + x_{microglia}}{x_{\max} A_{angiogenesis}}\right)\left(1 + \frac{u_{astro\_IL1} y_{IL1}}{s_{IL1} + y_{IL1}}\right)\left(1 + \frac{u_{astro\_PGE2} y_{PGE2}}{s_{PGE2} + y_{PGE2}}\right)$$

The first term of the right-hand side of Eq. (5) represents supply of astrocytes from progenitors. The second term is the result of proliferation minus mutation.

IL-1

Ameboid microglia, when activated, release significant quantities of IL-1 [76-78]. Astrocyte is observed to release IL-1 in culture [76,79]. Malignant glioma cells also secrete or express IL-1 [80-82].

In vitro, SCF inhibits microglial proliferation and their expression of the inflammatory cytokine IL-1β [69].

$$\dot{y}_{IL1} = k_{IL1\_glio} x_{glioma} + k_{IL1\_micro} x_{microglia} \frac{s_{SCF}}{s_{SCF} + y_{SCF}} + k_{IL1\_astro} x_{astrocyte} - d_{IL1} y_{IL1} \qquad (6)$$

IL-6

Glioma secretes IL-6 [29,83-86].

When cultured in the presence of IL-1β, the human glioma cell lines U251 and HP591 demonstrated a marked increase in IL-6 production [83].

IL-1β has been shown to exert a strong inducing signal for IL-6 in primary human/rat astrocytes [78,87].

IL-6 also released by microglia [78,88,89]

$$\dot{y}_{IL6} = k_{IL6\_glio} x_{glioma} \left(1 + \frac{u_{IL6\_IL1} y_{IL1}}{s_{IL1} + y_{IL1}}\right) + k_{IL6\_astro} x_{astrocyte} \left(\frac{y_{IL1}}{s_{IL1} + y_{IL1}}\right) + k_{IL6\_micro} x_{microglia} - d_{IL6} y_{IL6} \qquad (7)$$

IL-10

Microglia are the major source of IL-10 in gliomas [33,90].

$$\dot{y}_{IL10} = k_{IL10\_micro} x_{microglia} - d_{IL10} y_{IL10}$$
(8)

TNF-α

TNF-α is one of the products of activated microglia [30,78,91-93].

In vitro, SCF down-regulates microglial expression of TNF-α [69].

Astrocyte produces TNF-α in response to IL-1β [78,94-96].

$$\dot{y}_{TNF\alpha} = k_{TNF\alpha\_micro} x_{microglia} \frac{s_{SCF}}{s_{SCF} + y_{SCF}} + k_{TNF\alpha\_astro} x_{astrocyte} \left(\frac{y_{IL1}}{s_{IL1} + y_{IL1}} \right) - d_{TNF\alpha} y_{TNF\alpha} \quad (9)$$

TGF-β

The glioma cancer stem cells produce TGF-β1 [97].

Human GBM cell lines have been shown to produce TGF-β2 [31]. IL-1β also modulates the secretion of TGF-β from glioma cells in vitro, although the modulation has been shown to be both stimulatory and inhibitory, depending upon the cell line used [98,99].

Microglia has been shown to derive TGF-beta [100-102].

$$\dot{y}_{TGF\beta} = k_{TGF\beta\_ASC} x_{ASC} + k_{TGF\beta\_glio} x_{glioma} \left(1 + \frac{u_{TGF\beta\_IL1} y_{IL1}}{s_{IL1} + y_{IL1}} \right) + k_{TGF\beta\_micro} x_{microgla} - d_{TGF\beta} y_{TGF\beta}$$

(10)

EGF

EGF can be produced by activated microglia in vitro [103].

Heparin binding-EGF (Hb-EGF), a member of the EGF family, is produced by gliomas [104].

$$\dot{y}_{EGF} = k_{EGF\_glio} x_{glioma} + k_{EGF} x_{microglia} - d_{EGF} y_{EGF} \quad (11)$$

VEGF

Glioblastoma stem cells consistently secreted markedly elevated levels of VEGF [14,105].

Both microglia and gliomas secrete VEGF [12,13].

TNF-α increases VEGF expression in glioma cells in vitro [43].

MIF has been observed to induce a significant dose-dependent increase of VEGF [54,106].

$$\dot{y}_{VEGF} = k_{VEGF\_ASC} x_{ASC} + k_{VEGF\_glio} x_{glioma} \left(1 + \frac{u_{VEGF\_TNF\alpha} y_{TNF\alpha}}{s_{TNF\alpha} + y_{TNF\alpha}} \right) \left(1 + \frac{u_{VEGF\_MIF} y_{MIF}}{s_{MIF} + y_{MIF}} \right) + k_{VEGF\_micro} x_{microglia} - d_{VEGF} y_{VEGF} \quad (12)$$

FGF

FGF5 is frequently expressed in embryonic tissues and has been recently described as a stem cell marker [107]. Consequently, up-regulation during malignant progression might reflect dedifferentiation and acquisition of stem cell-like properties [5]. FGF has also been recognized as an autocrine signaling pathway in human embryonic stem cells [108].

Secreted FGF5 protein has been reported to generally present in the GBM cells *in vivo* and *in vitro* [5].

$$\dot{y}_{FGF} = k_{FGF\_ASC} x_{ASC} + k_{FGF\_glio} x_{glioma} - d_{FGF} y_{FGF} \tag{13}$$

HGF

HGF and its receptor, c-Met, have been found in microglia [65] and gliomas [44,109].

The expression of HGF in microglia is up-regulated by PGE2 in vitro and in vivo after ischemic injury [65].

$$\dot{y}_{HGF} = k_{HGF\_glio} x_{glioma} + k_{HGF\_micro} x_{microglia} \left(1 + \frac{u_{HGF\_PGE2} y_{PGE2}}{s_{PGE2} + y_{PGE2}}\right) - d_{HGF} y_{HGF} \tag{14}$$

MCP-1

Glioma cells express the microglia chemoattractant, MCP-1, at the mRNA and protein levels [58-60].

$$\dot{y}_{MCP1} = k_{MCP1\_glio} x_{glioma} - d_{MCP1} y_{MCP1} \tag{15}$$

MIF

MIF has been shown to be produced by glioma cell [110], and its expression was up-regulated under hypoxic and hypoglycemic stress conditions in vitro [10].

MIF was also secreted by activated microglia [20], and its secretion from macrophage can be induced by TNF-α [111].

$$\dot{y}_{MIF} = k_{MIF\_micro} x_{microglia} \left(1 + \frac{u_{MIF\_TNF\alpha} y_{TNF\alpha}}{s_{TNF\alpha} + y_{TNF\alpha}}\right) + k_{MIF\_glio} \left(1 + \frac{u_{MIF\_glio} x_{glioma}}{s_{glioma} + x_{glioma}}\right) x_{glioma} - d_{MIF} y_{MIF} \tag{16}$$

PGE2

Glioma-infiltrating microglia are a major source of PGE2 production through the COX-2 pathway [112].

$$\dot{y}_{PGE2} = k_{PGE2\_micro} x_{microglia} - d_{PGE2} y_{PGE2} \tag{17}$$

GM-CSF

Glioma cell lines express GM-CSF [47,113]. TGF-β2 and PGE2 has been shown to suppress GM-CSF production by gliomas in vitro [114]. IL-10 inhibits GM-CSF[115-117].

$$\dot{y}_{GMCSF} = k_{GMCSF\_glio} x_{glioma} \frac{s_{PGE2}}{s_{PGE2} + y_{PGE2}} \frac{s_{TGF\beta}}{s_{TGF\beta} + y_{TGF\beta}} + k_{GMCSF\_micro} x_{microglia} - d_{GMCSF} \left(1 + \frac{u_{GMCSF\_IL10} y_{IL10}}{s_{IL10} + y_{IL10}}\right) y_{GMCSF} \quad (18)$$

G-CSF

G-CSF is expressed constitutively in some glioma cell lines and in human gliomas [47,49]. IL-10 inhibits G-CSF[115,116].

$$\dot{y}_{GCSF} = k_{GCSF\_glio} x_{glioma} - d_{GCSF} \left(1 + \frac{u_{GCSF\_IL10} y_{IL10}}{s_{IL10} + y_{IL10}}\right) y_{GCSF}$$

(19)

SCF

SCF and its receptor c-kit, are highly expressed in glioma cell lines [50,51] and microglia [118,119].

$$\dot{y}_{SCF} = k_{SCF\_glio} x_{glioma} + k_{SCF\_micro} x_{microglia} - d_{SCF} y_{SCF} \quad (20)$$

2. Stochastic description of rate parameters

2.1 Bounded noise

We use bounded noise to describe the stochastic proliferation / mutation / differentiation /dedifferentiation rate (r_{ASC}, r_{glioma}, $r_{astrocyte}$, $r_{microglia}$, p_{glio_astro}, p_{glio_ASC}, p_{ASC_glio}).

$$r_{stochastic}(t)=r_{deterministic}(1+\varepsilon\sin(\Omega t+\sigma W(t)+\Delta)) \quad (21)$$

where $W(t)$ is a standard Wiener process. $\zeta(t) = \varepsilon\sin(\Omega t+\sigma W(t)+\Delta)$ is the so called bounded noise with the mathematical expectation at a fixed time t

$$E[\zeta(t)] = e^{-t\sigma^2/2}\sin(\Omega t + \Delta) = \begin{cases} 0 & \sigma \to \infty \\ \sin(\Omega t + \Delta) & \sigma \to 0 \end{cases} \quad (22)$$

and the auto correlation function

$$R(\tau) = \frac{1}{2}\exp\left(-\frac{\sigma^2}{2}|\tau|\right)\cos\Omega\tau = \begin{cases} \frac{1}{2}\delta_{\tau,0} & \sigma \to \infty \\ \frac{1}{2}\cos\Omega\tau & \sigma \to 0 \end{cases}$$

(23)

where $\delta_{\tau,0}$ is Kronecker delta. Thus, the bounded noise $\zeta(t)$ tends to a finite power white noise as $\sigma \to \infty$, and becomes a harmonic noise as $\sigma \to 0$.

The stochastic rate term $r_{\text{stochastic}}(t)$ describes a stochastic fluctuation around the average rate $r_{\text{deterministic}}$, which should be estimated according to the experimental data. There are three critical parameters: the strength factor $0 \le \varepsilon < 1$, the bandwidth factor $\sigma \ge 0$, and the center frequency $\Omega > 0$.

The flexible and adjustable characteristics of bounded noise make it an appropriate description of the intrinsically random rates and a good approximation to cell cycles according to heterogeneous scenarios.

Cell cycle is obviously periodic; however, the endogenous and exogenous signals that influence the cellular activity may be aperiodic. Thus, it is reasonable to assume that the rate is a stochastic perturbation to periodic fluctuations. In case the cellular activity observed in experiment shows regular periodic fluctuations around a mean value, a small σ should be adopted. Then, the center frequency Ω is determined by the period of cell cycle $T_{\text{cell cycle}}$

$$\Omega = \frac{2\pi}{T_{\text{cell cycle}}} = \frac{2\pi \times (\text{basal proliferation rate})}{\ln 2} \tag{24}$$

Alternatively, when there do not exist regular fluctuations or a characteristic frequency band, a large σ will be chosen to capture the stochastic nature.

2.2 Poisson white noise

We introduce Poisson white noise $\xi(t)$ to describe the stochastic immigration, emigration and supply from normal neural stem cell / monocytes / progenitors (c_{QSC}, $c_{\text{microglia}}$, $c_{\text{astrocyte}}$).

$$\xi(t) = \sum_{k=1}^{N(t)} Y_k \delta(t - \tau_k) \tag{25}$$

$\xi(t)$ is the stochastic representation of discrete event type fluctuation. Y_k is the magnitude of kth discrete event, i.e., the number of cell increasing (decreasing) at time point $t = \tau_k$, $N_i(t)$ denotes a non-homogeneous Poisson counting process with arrival rate function $\lambda_i(t) > 0$ (i.e., the number of events per unit time) and gives the number of events that arrive in the time interval $[0,t]$.

$$\lambda_{QSC}(t) = c_{QSC} \tag{26}$$

$$\lambda_{astrocyte}(t) = c_{astrocyte} \tag{27}$$

$$\lambda_{microglia}(t) = c_{microglia}\left(1 + \frac{u_{micro\_MCP1} y_{MCP1}}{s_{MCP1} + y_{MCP1}}\right) \tag{28}$$

2.3 Gaussian white noise

We use Gaussian white noise to describe the stochastic fluctuation of cytokine secretion rates and up-regulation ratio via receptor kinase signaling (k_i and u_i).

$$k_{\text{stochastic}}(t) = k_{\text{deterministic}} \max\left(0, 1 + \sigma_g \eta(t)\right) \tag{29}$$

$$u_{\text{stochastic}}(t) = u_{\text{deterministic}} \max\left(0, 1 + \sigma_g \eta(t)\right) \tag{30}$$

where $\eta(t)$ is a Gaussian white noise with mean zero and standard deviation 1.

3. Sensitivity analysis

To systematically evaluate the influence of each cytokine on tumorigenesis rate, we conducted a sensitivity test, in which the sensitivity factor of cytokine x_i can be calculated as

$$S_i \equiv \left.\frac{\partial F(\mathbf{x})}{\partial x_i}\right|_{\mathbf{x}=\mathbf{x}^0} \tag{31}$$

where $F(\mathbf{x})$ is the objective function (e.g. tumorigenesis time, cell density, cytokine concentration), and $\mathbf{x}^0$ is the local parameter profile. The sensitivity factor S_i can be calculated numerically. The scenarios are dependent on the choice of objective function F. In the context, we studied the parameter sensitivity for tumorigenesis time and therapeutic effect, respectively. The definition is also local-state-dependent, that is, each sensitivity factor is calculated locally at parameter profile $\mathbf{x}^0$. Thus, we showed inter-patient heterogeneity by comparison of the sensitivity analyses results with diverse individual patient profiles, and further designed patient-based cytokine-targeted therapy.

The results show marked effects of these cytokines on the development of glioma and suggests and possibility of designing therapeutic intervention by targeting cytokine signaling pathways (both cytokine production and receptor expression level) (Supplementary Fig. S9). It was also found that the quantitative results are context specific; the exact time for observing tumor formation ($1\text{x}10^6$ cells/ml) depends on the profile of all initial parameters for each patient (Supplementary Tables S3 and S4). The greater the difference between cytokine sensitivity factor landscapes, the greater the inter-patients heterogeneity. In addition to quantitative manifestation of inter-patient heterogeneity, sensitivity analysis also points to a venue to identify a cytokine profile that potentially can serve as molecule signature for tumor sub-classification, and thus provides a means to stratify patients via their cytokine profiles and to design individualized treatment.

References

1 Greco, V. *et al.* A Two-Step Mechanism for Stem Cell Activation during Hair Regeneration. *Cell Stem Cell* **4**, 155-169, doi:10.1016/j.stem.2008.12.009 (2009).

2 Kelly, J. J. P. *et al.* Proliferation of Human Glioblastoma Stem Cells Occurs Independently of Exogenous Mitogens. *Stem Cells* **27**, 1722-1733, doi:10.1002/stem.98 (2009).

3 Hermanson, O., Jepsen, K. & Rosenfeld, M. G. N-CoR controls differentiation of neural stem cells into astrocytes. *Nature* **419**, 934-939, doi:10.1038/nature01156 (2002).

4 Wang, H. *et al.* Targeting Interleukin 6 Signaling Suppresses Glioma Stem Cell Survival and Tumor Growth. *Stem Cells* **27**, 2393-2404, doi:10.1002/stem.188 (2009).

5 Allerstorfer, S. *et al.* FGF5 as an oncogenic factor in human glioblastoma multiforme: autocrine and paracrine activities. *Oncogene* **27**, 4180-4190, doi:10.1038/onc.2008.61 (2008).

6 Schmidt, N. O. *et al.* Levels of vascular endothelial growth factor, hepatocyte growth factor scatter factor and basic fibroblast growth factor in human gliomas and their relation to angiogenesis. *International Journal of Cancer* **84**, 10-18 (1999).

7 Sunderkotter, C., Steinbrink, K., Goebeler, M., Bhardwaj, R. & Sorg, C. MACROPHAGES AND ANGIOGENESIS. *Journal of Leukocyte Biology* **55**, 410-422 (1994).

8 Voronov, E. *et al.* IL-1 is required for tumor invasiveness and angiogenesis. *Proceedings of the National Academy of Sciences of the United States of America* **100**, 2645-2650, doi:10.1073/pnas.0437939100 (2003).

9 Abounader, R. & Laterra, J. Scatter factor/hepatocyte growth factor in brain tumor growth and angiogenesis. *Neuro-Oncology* **7**, 436-451, doi:10.1215/s1152851705000050 (2005).

10 Bacher, M. *et al.* Up-regulation of macrophage migration inhibitory factor gene and protein expression in glial tumor cells during hypoxic and hypoglycemic stress indicates a critical role for angiogenesis in glioblastoma multiforme. *American Journal of Pathology* **162**, 11-17 (2003).

11 Saijo, Y. *et al.* Proinflammatory cytokine IL-1 beta promotes tumor growth of Lewis lung carcinoma by induction of angiogenic factors: In vivo analysis of tumor-stromal interaction. *Journal of Immunology* **169**, 469-475 (2002).

12 Tsai, J. C., Goldman, C. K. & Gillespie, G. Y. VASCULAR ENDOTHELIAL GROWTH-FACTOR IN HUMAN GLIOMA CELL-LINES - INDUCED SECRETION BY EGF, PDGF-BB, AND BFGF. *J. Neurosurg.* **82**, 864-873 (1995).

13 Lafuente, J. V. *et al.* Expression of vascular endothelial growth factor (VEGF) and platelet-derived growth factor receptor-beta (PDGFR-beta) in human gliomas. *Journal of Molecular Neuroscience* **13**, 177-185 (1999).

14 Yao, X. H. *et al.* Glioblastoma stem cells produce vascular endothelial growth factor by activation of a G-protein coupled formylpeptide receptor FPR. *Journal of Pathology* **215**, 369-376, doi:10.1002/path.2356 (2008).

15 Stefanik, D. F., Rizkalla, L. R., Soi, A., Goldblatt, S. A. & Rizkalla, W. M. ACIDIC AND BASIC FIBROBLAST GROWTH-FACTORS ARE PRESENT IN GLIOBLASTOMA-MULTIFORME. *Cancer Research* **51**, 5760-5765 (1991).

16 Takahashi, J. A. *et al.* CORRELATION OF BASIC FIBROBLAST GROWTH-FACTOR EXPRESSION LEVELS WITH THE DEGREE OF MALIGNANCY AND VASCULARITY IN HUMAN GLIOMAS. *J. Neurosurg.* **76**, 792-798 (1992).

17 Stan, A. C., Nemati, M. N., Pietsch, T., Walter, G. F. & Dietz, H. IN-VIVO INHIBITION OF ANGIOGENESIS AND GROWTH OF THE HUMAN U-87 MALIGNANT GLIAL TUMOR BY TREATMENT WITH AN ANTIBODY AGAINST BASIC FIBROBLAST GROWTH-FACTOR. *J. Neurosurg.* **82**, 1044-1052 (1995).

18 Dunn, I. F., Heese, O. & Black, P. M. Growth factors in glioma angiogenesis: FGFs, PDGF, EGF, and TGFs. *Journal of Neuro-Oncology* **50**, 121-137 (2000).
19 Krockenberger, M. *et al.* Macrophage migration inhibitory factor contributes to the immune escape of ovarian cancer by down-regulating NKG2D. *Journal of Immunology* **180**, 7338-7348 (2008).
20 Bach, J. P. *et al.* The Role of Macrophage Inhibitory Factor in Tumorigenesis and Central Nervous System Tumors. *Cancer* **115**, 2031-2040, doi:10.1002/cncr.24245 (2009).
21 Sun, L. X. *et al.* Neuronal and glioma-derived stem cell factor induces angiogenesis within the brain. *Cancer Cell* **9**, 287-300, doi:10.1016/j.ccr.2006.03.003 (2006).
22 Fan, X. L., Salford, L. G. & Widegren, B. Glioma stem cells: Evidence and limitation. *Seminars in Cancer Biology* **17**, 214-218, doi:10.1016/j.semcancer.2006.04.002 (2007).
23 Holland, E. C., Hively, W. P., DePinho, R. A. & Varmus, H. E. A constitutively active epidermal growth factor receptor cooperates with disruption of G(1) cell-cycle arrest pathways to induce glioma-like lesions in mice. *Genes Dev.* **12**, 3675-3685 (1998).
24 Engebraaten, O., Bjerkvig, R., Pedersen, P. H. & Laerum, O. D. EFFECTS OF EGF, BFGF, NGF AND PDGF(BB) ON CELL PROLIFERATIVE, MIGRATORY AND INVASIVE CAPACITIES OF HUMAN BRAIN-TUMOR BIOPSIES INVITRO. *International Journal of Cancer* **53**, 209-214 (1993).
25 Loilome, W. *et al.* Glioblastoma cell growth is suppressed by disruption of fibroblast growth factor pathway signaling. *Journal of Neuro-Oncology* **94**, 359-366, doi:10.1007/s11060-009-9885-5 (2009).
26 Dancey, J. E. & Freidlin, B. Targeting epidermal growth factor receptor - are we missing the mark? *Lancet* **362**, 62-64 (2003).
27 Gadji, M. *et al.* EGF receptor inhibitors in the treatment of glioblastoma multiform: Old clinical allies and newly emerging therapeutic concepts. *European Journal of Pharmacology* **625**, 23-30, doi:10.1016/j.ejphar.2009.10.010 (2009).
28 Loew, S., Schmidt, U., Unterberg, A. & Halatsch, M. E. The Epidermal Growth Factor Receptor as a Therapeutic Target in Glioblastoma Multiforme and other Malignant Neoplasms. *Anti-Cancer Agents Med. Chem.* **9**, 703-715 (2009).
29 Goswami, S., Gupta, A. & Sharma, S. K. Interleukin-6-mediated autocrine growth promotion in human glioblastoma multiforme cell line U87MG. *Journal of Neurochemistry* **71**, 1837-1845 (1998).
30 Hao, C. H. *et al.* Cytokine and cytokine receptor mRNA expression in human glioblastomas: evidence of Th1, Th2 and Th3 cytokine dysregulation. *Acta Neuropathol.* **103**, 171-178 (2002).
31 Constam, D. B. *et al.* DIFFERENTIAL EXPRESSION OF TRANSFORMING GROWTH FACTOR-BETA-1, FACTOR-BETA-2, AND FACTOR-BETA-3 BY GLIOBLASTOMA CELLS, ASTROCYTES, AND MICROGLIA. *Journal of Immunology* **148**, 1404-1410 (1992).
32 Olofsson, A. *et al.* TRANSFORMING GROWTH FACTOR-BETA-1, FACTOR-BETA-2, AND FACTOR-BETA-3 SECRETED BY A HUMAN GLIOBLASTOMA CELL-LINE - IDENTIFICATION OF SMALL AND DIFFERENT FORMS OF LARGE LATENT COMPLEXES. *Journal of Biological Chemistry* **267**, 19482-19488 (1992).
33 Huettner, C., Czub, S., Kerkau, S., Roggendorf, W. & Tonn, J. C. Interleukin 10 is expressed in human gliomas in vivo and increases glioma cell proliferation and motility in vitro. *Anticancer Research* **17**, 3217-3224 (1997).
34 Libermann, T. A. *et al.* AMPLIFICATION AND OVEREXPRESSION OF THE EGF RECEPTOR GENE IN PRIMARY HUMAN GLIOBLASTOMAS. *Journal of Cell Science*, 161-172 (1985).
35 Helseth, E. *et al.* Amplification of the epidermal growth factor receptor gene in biopsy specimens from human intracranial tumours. *Br J Neurosurg* **2**, 217-225 (1988).

36 Steck, P. A., Lee, P., Hung, M. C. & Yung, W. K. A. EXPRESSION OF AN ALTERED EPIDERMAL GROWTH-FACTOR RECEPTOR BY HUMAN GLIOBLASTOMA CELLS. *Cancer Research* **48**, 5433-5439 (1988).

37 Andersson, A., Holmberg, A., Carlsson, J., Ponten, J. & Westermark, B. BINDING OF EPIDERMAL GROWTH FACTOR-DEXTRAN CONJUGATES TO CULTURED GLIOMA-CELLS. *International Journal of Cancer* **47**, 439-444 (1991).

38 Prahl, M., Nederman, T., Carlsson, J. & Sjodin, L. BINDING OF EPIDERMAL GROWTH-FACTOR (EGF) TO A CULTURED HUMAN GLIOMA CELL-LINE. *Journal of Receptor Research* **11**, 791-812 (1991).

39 Torp, S. H., Helseth, E., Dalen, A. & Unsgaard, G. EPIDERMAL GROWTH-FACTOR RECEPTOR EXPRESSION IN HUMAN GLIOMAS. *Cancer Immunology Immunotherapy* **33**, 61-64 (1991).

40 Kleihues, P., Soylemezoglu, F., Schauble, B., Scheithauer, B. W. & Burger, P. C. HISTOPATHOLOGY, CLASSIFICATION, AND GRADING OF GLIOMAS. *Glia* **15**, 211-221 (1995).

41 Adachi, K. *et al.* ENHANCEMENT OF EPIDERMAL GROWTH-FACTOR RECEPTOR EXPRESSION ON GLIOMA-CELLS BY RECOMBINANT TUMOR-NECROSIS-FACTOR-ALPHA. *Cancer Immunology Immunotherapy* **34**, 370-376 (1992).

42 Kunkel, P. *et al.* Inhibition of glioma angiogenesis and growth in vivo by systemic treatment with a monoclonal antibody against vascular endothelial growth factor receptor-2. *Cancer Research* **61**, 6624-6628 (2001).

43 Ryuto, M. *et al.* Induction of vascular endothelial growth factor by tumor necrosis factor alpha in human glioma cells - Possible roles of SP-1. *Journal of Biological Chemistry* **271**, 28220-28228 (1996).

44 Kunkel, P. *et al.* Expression and localization of scatter factor/hepatocyte growth factor in human astrocytomas. *Neuro-Oncology* **3**, 82-88 (2001).

45 Jeffers, M., Rong, S. & VandeWoude, G. F. Hepatocyte growth factor scatter factor Met signaling in tumorigenicity and invasion/metastasis. *Journal of Molecular Medicine-Jmm* **74**, 505-513 (1996).

46 Koochekpour, S. *et al.* Met and hepatocyte growth factor scatter factor expression in human gliomas. *Cancer Research* **57**, 5391-5398 (1997).

47 Nitta, T. *et al.* EXPRESSION OF GRANULOCYTE COLONY STIMULATING FACTOR AND GRANULOCYTE-MACROPHAGE COLONY STIMULATING FACTOR GENES IN HUMAN ASTROCYTOMA CELL-LINES AND IN GLIOMA SPECIMENS. *Brain Research* **571**, 19-25 (1992).

48 Tada, M., Diserens, A. C., Desbaillets, I. & Detribolet, N. ANALYSIS OF CYTOKINE RECEPTOR MESSENGER-RNA EXPRESSION IN HUMAN GLIOBLASTOMA CELLS AND NORMAL ASTROCYTES BY REVERSE-TRANSCRIPTION POLYMERASE CHAIN-REACTION. *J. Neurosurg.* **80**, 1063-1073 (1994).

49 Mueller, M. M. *et al.* Autocrine growth regulation by granulocyte colony-stimulating factor and granulocyte macrophage colony-stimulating factor in human gliomas with tumor progression. *American Journal of Pathology* **155**, 1557-1567 (1999).

50 Stanulla, M., Welte, K., Hadam, M. R. & Pietsch, T. COEXPRESSION OF STEM-CELL FACTOR AND ITS RECEPTOR C-KIT IN HUMAN-MALIGNANT GLIOMA CELL-LINES. *Acta Neuropathol.* **89**, 158-165 (1995).

51 Hamel, W. & Westphal, M. The road less travelled: c-kit and stem cell factor. *Journal of Neuro-Oncology* **35**, 327-333 (1997).

52 Berdel, W. E. *et al.* RECOMBINANT HUMAN STEM-CELL FACTOR STIMULATES GROWTH OF A HUMAN GLIOBLASTOMA CELL-LINE EXPRESSING C-KIT PROTOONCOGENE. *Cancer Research* **52**, 3498-3502 (1992).

53 Takahashi, N. *et al.* Involvement of macrophage migration inhibitory factor (MIF) in the mechanism of tumor cell growth. *Molecular Medicine* **4**, 707-714 (1998).

54 Ren, Y. *et al.* Upregulation of macrophage migration inhibitory factor contributes to induced N-Myc expression by the activation of ERK signaling pathway and increased expression of interleukin-8 and VEGF in neuroblastoma. *Oncogene* **23**, 4146-4154, doi:10.1038/sj.onc.1207490 (2004).

55 Fingerle-Rowson, G. *et al.* The p53-dependent effects of macrophage migration inhibitory factor revealed by gene targeting. *Proceedings of the National Academy of Sciences of the United States of America* **100**, 9354-9359, doi:10.1073/pnas.1533295100 (2003).

56 Westphal, M., Neuss, M. & Herrmann, H. D. PROSTAGLANDINS - ANTIPROLIFERATIVE EFFECT OF PGD2 ON CULTURED HUMAN GLIOMA-CELLS. *Acta Neurochirurgica* **83**, 56-61 (1986).

57 Guillemin, G. J. & Brew, B. J. Microglia, macrophages, perivascular macrophages, and pericytes: a review of function and identification. *Journal of Leukocyte Biology* **75**, 388-397, doi:10.1189/jlb.0303114 (2004).

58 Leung, S. Y., Wong, M. P., Chung, L. P., Chan, A. S. Y. & Yuen, S. T. Monocyte chemoattractant protein-1 expression and macrophage infiltration in gliomas. *Acta Neuropathol.* **93**, 518-527 (1997).

59 Prat, E. *et al.* The human astrocytoma cell line U373MG produces monocyte chemotactic protein (MCP)-1 upon stimulation with beta-amyloid protein. *Neurosci. Lett.* **283**, 177-180 (2000).

60 Takeshima, H., Kuratsu, J. I., Takeya, M., Yoshimura, T. & Ushio, Y. EXPRESSION AND LOCALIZATION OF MESSENGER-RNA AND PROTEIN FOR MONOCYTE CHEMOATTRACTANT PROTEIN-1 IN HUMAN-MALIGNANT GLIOMA. *J. Neurosurg.* **80**, 1056-1062 (1994).

61 Galasso, J. M. *et al.* Experimental gliosarcoma induces chemokine receptor expression in rat brain. *Experimental Neurology* **161**, 85-95 (2000).

62 Platten, M. *et al.* Monocyte chemoattractant protein-1 increases microglial infiltration and aggressiveness of gliomas. *Annals of Neurology* **54**, 388-392, doi:10.1002/ana.10679 (2003).

63 Nolte, C., Kirchhoff, F. & Kettenmann, H. Epidermal growth factor is a motility factor for microglial cells in vitro: Evidence for EGF receptor expression. *European Journal of Neuroscience* **9**, 1690-1698 (1997).

64 Forstreuter, F., Lucius, R. & Mentlein, R. Vascular endothelial growth factor induces chemotaxis and proliferation of microglial cells. *Journal of Neuroimmunology* **132**, 93-98 (2002).

65 Zhang, L. L., Himi, T. & Murota, S. I. Induction of hepatocyte growth factor (HGF) in rat microglial cells by prostaglandin E-2. *Journal of Neuroscience Research* **62**, 389-395 (2000).

66 Badie, B., Schartner, J., Klaver, J. & Vorpahl, J. In vitro modulation of microglia motility by glioma cells is mediated by hepatocyte growth factor scatter factor. *Neurosurgery* **44**, 1077-1082 (1999).

67 Giulian, D. & Ingeman, J. E. COLONY-STIMULATING FACTORS AS PROMOTERS OF AMEBOID MICROGLIA. *Journal of Neuroscience* **8**, 4707-4717 (1988).

68 Suzumura, A., Sawada, M., Yamamoto, H. & Marunouchi, T. TRANSFORMING GROWTH-FACTOR-BETA SUPPRESSES ACTIVATION AND PROLIFERATION OF MICROGLIA IN-VITRO. *Journal of Immunology* **151**, 2150-2158 (1993).

69 Zhang, S. C. & Fedoroff, S. Modulation of microglia by stem cell factor. *Journal of Neuroscience Research* **53**, 29-37 (1998).

70 Suzuki, S. O. & Goldman, J. E. Multiple cell populations in the early postnatal subventricular zone take distinct migratory pathways: A dynamic study of glial and neuronal progenitor migration. *Journal of Neuroscience* **23**, 4240-4250 (2003).

71 Giulian, D. & Lachman, L. B. INTERLEUKIN-1 STIMULATION OF ASTROGLIAL PROLIFERATION AFTER BRAIN INJURY. *Science* **228**, 497-499 (1985).

72 Giulian, D., Young, D. G., Woodward, J., Brown, D. C. & Lachman, L. B. INTERLEUKIN-1 IS AN ASTROGLIAL GROWTH-FACTOR IN THE DEVELOPING BRAIN. *Journal of Neuroscience* **8**, 709-714 (1988).

73 Giulian, D. & Baker, T. J. PEPTIDES RELEASED BY AMEBOID MICROGLIA REGULATE ASTROGLIAL PROLIFERATION. *Journal of Cell Biology* **101**, 2411-2415 (1985).

74 Cuny, E. *et al.* Association of elevated glial expression of interleukin-1 beta with improved survival in patients with glioblastomas multiforme. *J. Neurosurg.* **96**, 294-301 (2002).

75 Zhang, D. *et al.* Prostaglandin E2 released from activated microglia enhances astrocyte proliferation in vitro. *Toxicology and Applied Pharmacology* **238**, 64-70, doi:10.1016/j.taap.2009.04.015 (2009).

76 Giulian, D., Baker, T. J., Shih, L. C. N. & Lachman, L. B. INTERLEUKIN-1 OF THE CENTRAL-NERVOUS-SYSTEM IS PRODUCED BY AMEBOID MICROGLIA. *Journal of Experimental Medicine* **164**, 594-604 (1986).

77 Giulian, D. & Baker, T. J. CHARACTERIZATION OF AMEBOID MICROGLIA ISOLATED FROM DEVELOPING MAMMALIAN BRAIN. *Journal of Neuroscience* **6**, 2163-2178 (1986).

78 Lee, S. C., Liu, W., Dickson, D. W., Brosnan, C. F. & Berman, J. W. CYTOKINE PRODUCTION BY HUMAN FETAL MICROGLIA AND ASTROCYTES - DIFFERENTIAL INDUCTION BY LIPOPOLYSACCHARIDE AND IL-1-BETA. *Journal of Immunology* **150**, 2659-2667 (1993).

79 Fontana, A., Kristensen, F., Dubs, R., Gemsa, D. & Weber, E. PRODUCTION OF PROSTAGLANDIN-E AND AN INTERLEUKIN-1 LIKE FACTOR BY CULTURED ASTROCYTES AND C6 GLIOMA-CELLS. *Journal of Immunology* **129**, 2413-2419 (1982).

80 Fontana, A., Hengartner, H., Detribolet, N. & Weber, E. GLIOBLASTOMA CELLS RELEASE INTERLEUKIN-1 AND FACTORS INHIBITING INTERLEUKIN-2-MEDIATED EFFECTS. *Journal of Immunology* **132**, 1837-1844 (1984).

81 Gauthier, T. *et al.* EXPRESSION AND RELEASE OF INTERLEUKIN-1 BY HUMAN GLIOBLASTOMA CELLS-INVITRO AND INVIVO. *Acta Neurochirurgica* **121**, 199-205 (1993).

82 Lee, J. C., Simon, P. L. & Young, P. R. CONSTITUTIVE AND PMA-INDUCED INTERLEUKIN-1 PRODUCTION BY THE HUMAN ASTROCYTOMA CELL-LINE T24. *Cellular Immunology* **118**, 298-311 (1989).

83 Cinque, S., Willems, J., Depraetere, S., Vermeire, L. & Joniau, M. INVITRO EFFECT OF INTERLEUKIN-1-BETA ON HUMAN GLIOMA CELL-LINES - REGULATION OF CELL-PROLIFERATION AND IL-6 PRODUCTION. *Immunology Letters* **34**, 267-271 (1992).

84 Nitta, T. & Sato, K. Expression of interleukin-6 gene in human astrocyte cell lineages. *J Clin Neurosci* **1**, 53-57 (1994).

85 Van Meir, E., Sawamura, Y., Diserens, A. C., Hamou, M. F. & Detribolet, N. HUMAN GLIOBLASTOMA CELLS RELEASE INTERLEUKIN-6 INVIVO AND INVITRO. *Cancer Research* **50**, 6683-6688 (1990).

86 Yamanaka, R., Tanaka, R. & Yoshida, S. Effects of irradiation on cytokine production in glioma cells lines. *Neurologia Medico-Chirurgica* **33**, 744-748 (1993).

87 Benveniste, E. N., Sparacio, S. M., Norris, J. G., Grenett, H. E. & Fuller, G. M. INDUCTION AND REGULATION OF INTERLEUKIN-6 GENE-EXPRESSION IN RAT ASTROCYTES. *Journal of Neuroimmunology* **30**, 201-212 (1990).

88 Jang, S., Kelley, K. W. & Johnson, R. W. Luteolin reduces IL-6 production in microglia by inhibiting JNK phosphorylation and activation of AP-1. *Proceedings of the National Academy of Sciences of the United States of America* **105**, 7534-7539, doi:10.1073/pnas.0802865105 (2008).

89 Nakanishi, M. *et al.* Microglia-derived interleukin-6 and leukaemia inhibitory factor promote astrocytic differentiation of neural stem/progenitor cells. *European Journal of Neuroscience* **25**, 649-658, doi:10.1111/j.1460-9568.2007.05309.x (2007).

90 Wagner, S. *et al.* Microglial macrophage expression of interleukin 10 in human glioblastomas. *International Journal of Cancer* **82**, 12-16 (1999).

91 Roessler, K. *et al.* DETECTION OF TUMOR NECROSIS FACTOR-EX PROTEIN AND MESSENGER-RNA IN HUMAN GLIAL BRAIN-TUMORS - COMPARISON OF IMMUNOHISTOCHEMISTRY WITH IN-SITU HYBRIDIZATION USING MOLECULAR PROBES. *J. Neurosurg.* **83**, 291-297 (1995).

92 Martin, F. C., Anton, P. A., Gornbein, J. A., Shanahan, F. & Merrill, J. E. PRODUCTION OF INTERLEUKIN-1 BY MICROGLIA IN RESPONSE TO SUBSTANCE-P - ROLE FOR A NONCLASSICAL NK-1 RECEPTOR. *Journal of Neuroimmunology* **42**, 53-60 (1993).

93 Lubernarod, J., Kage, R. & Leeman, S. E. SUBSTANCE-P ENHANCES THE SECRETION OF TUMOR-NECROSIS-FACTOR-ALPHA FROM NEUROGLIAL CELLS STIMULATED WITH LIPOPOLYSACCHARIDE. *Journal of Immunology* **152**, 819-824 (1994).

94 Chung, I. Y., Norris, J. G. & Benveniste, E. N. DIFFERENTIAL TUMOR-NECROSIS-FACTOR-ALPHA EXPRESSION BY ASTROCYTES FROM EXPERIMENTAL ALLERGIC ENCEPHALOMYELITIS-SUSCEPTIBLE AND ENCEPHALOMYELITIS-RESISTANT RAT STRAINS. *Journal of Experimental Medicine* **173**, 801-811 (1991).

95 Chung, I. Y. & Benveniste, E. N. TUMOR NECROSIS FACTOR-ALPHA PRODUCTION BY ASTROCYTES - INDUCTION BY LIPOPOLYSACCHARIDE, IFN-GAMMA, AND IL-1-BETA. *Journal of Immunology* **144**, 2999-3007 (1990).

96 Kim, H. M. *et al.* Inhibition of tumour necrosis factor-alpha secretion from rat astrocytes by Sesim-Tang. *Phytotherapy Research* **16**, 28-32 (2002).

97 Wu, A. *et al.* Glioma cancer stem cells induce immunosuppressive macrophages/microglia. *Neuro-Oncology* **12**, 1113-1125, doi:10.1093/neuonc/noq082 (2010).

98 Naganuma, H. *et al.* Modulation of transforming growth factor-beta secretion from malignant glioma cells by interleukin-1-beta. *Neurologia Medico-Chirurgica* **36**, 145-150, 189 (1996).

99 Naganuma, H. *et al.* Down-regulation of transforming growth factor-beta and interleukin-10 secretion from malignant glioma cells by cytokines and anticancer drugs. *Journal of Neuro-Oncology* **39**, 227-236 (1998).

100 Lindholm, D., Castren, E., Kiefer, R., Zafra, F. & Thoenen, H. TRANSFORMING GROWTH FACTOR-BETA-1 IN THE RAT-BRAIN - INCREASE AFTER INJURY AND INHIBITION OF ASTROCYTE PROLIFERATION. *Journal of Cell Biology* **117**, 395-400 (1992).

101 Kiefer, R., Supler, M. L., Toyka, K. V. & Streit, W. J. IN-SITU DETECTION OF TRANSFORMING GROWTH-FACTOR-BETA MESSENGER-RNA IN EXPERIMENTAL RAT GLIOMA AND REACTIVE GLIAL-CELLS. *Neurosci. Lett.* **166**, 161-164 (1994).

102 Wesolowska, A. *et al.* Microglia-derived TGF-beta as an important regulator of glioblastoma invasion - an inhibition of TGF-beta-dependent effects by shRNA against human TGF-beta type II receptor. *Oncogene* **27**, 918-930, doi:10.1038/sj.onc.1210683 (2008).

103 Briers, T. W., Desmaretz, C. & Vanmechelen, E. GENERATION AND CHARACTERIZATION OF MOUSE MICROGLIAL CELL-LINES. *Journal of Neuroimmunology* **52**, 153-164 (1994).

104 Mishima, K. *et al.* Heparin-binding epidermal growth factor-like growth factor stimulates mitogenic signaling and is highly expressed in human malignant gliomas. *Acta Neuropathol.* **96**, 322-328 (1998).

105 Bao, S. D. *et al.* Stem cell-like glioma cells promote tumor angiogenesis through vascular endothelial growth factor. *Cancer Research* **66**, 7843-7848, doi:10.1158/0008-5472.can-06-1010 (2006).

106 Munaut, C., Boniver, J., Foidart, J. M. & Deprez, M. Macrophage migration inhibitory factor (MIF) expression in human glioblastomas correlates with vascular endothelial growth factor (VEGF) expression. *Neuropathology and Applied Neurobiology* **28**, 452-460 (2002).

107 Pelton, T. A., Sharma, S., Schulz, T. C., Rathjen, J. & Rathjen, P. D. Transient pluripotent cell populations during primitive ectoderm formation: correlation of in vivo and in vitro pluripotent cell development. *Journal of Cell Science* **115**, 329-339 (2002).

108 Dvorak, P. *et al.* Expression and potential role of fibroblast growth factor 2 and its receptors in human embryonic stem cells. *Stem Cells* **23**, 1200-1211, doi:10.1634/stemcells.2005-0303 (2005).

109 Yamada, T. *et al.* IMMUNOHISTOCHEMISTRY WITH ANTIBODIES TO HEPATOCYTE GROWTH-FACTOR AND ITS RECEPTOR PROTEIN (C-MET) IN HUMAN BRAIN-TISSUES. *Brain Research* **637**, 308-312 (1994).

110 Piette, C. *et al.* The Dexamethasone-induced Inhibition of Proliferation, Migration, and Invasion in Glioma Cell Lines Is Antagonized by Macrophage Migration Inhibitory Factor (MIF) and Can Be Enhanced by Specific MIF Inhibitors. *Journal of Biological Chemistry* **284**, 32483-32492, doi:10.1074/jbc.M109.014589 (2009).

111 Calandra, T., Bernhagen, J., Mitchell, R. A. & Bucala, R. MACROPHAGE IS AN IMPORTANT AND PREVIOUSLY UNRECOGNIZED SOURCE OF MACROPHAGE-MIGRATION INHIBITORY FACTOR. *Journal of Experimental Medicine* **179**, 1895-1902 (1994).

112 Badie, B. *et al.* Microglia cyclooxygenase-2 activity in experimental gliomas: Possible role in cerebral edema formation. *Clin. Cancer Res.* **9**, 872-877 (2003).

113 Yamanaka, R., Tanaka, R., Saitoh, T. & Okoshi, S. CYTOKINE GENE-EXPRESSION ON GLIOMA CELL-LINES AND SPECIMENS. *Journal of Neuro-Oncology* **21**, 243-247 (1994).

114 Frei, K. *et al.* GRANULOCYTE-MACROPHAGE COLONY-STIMULATING FACTOR (GM-CSF) PRODUCTION BY GLIOBLASTOMA CELLS - DESPITE THE PRESENCE OF INDUCING SIGNALS GM-CSF IS NOT EXPRESSED INVIVO. *Journal of Immunology* **148**, 3140-3146 (1992).

115 Malefyt, R. D., Abrams, J., Bennett, B., Figdor, C. G. & Devries, J. E. INTERLEUKIN-10(IL-10) INHIBITS CYTOKINE SYNTHESIS BY HUMAN MONOCYTES - AN AUTOREGULATORY ROLE OF IL-10 PRODUCED BY MONOCYTES. *Journal of Experimental Medicine* **174**, 1209-1220 (1991).

116 Moore, K. W., Malefyt, R. D., Coffman, R. L. & O'Garra, A. Interleukin-10 and the interleukin-10 receptor. *Annual Review of Immunology* **19**, 683-765 (2001).

117 Thomassen, M. J. *et al.* Elevated IL-10 inhibits GM-CSF synthesis in pulmonary alveolar proteinosis. *Autoimmunity* **36**, 285-290, doi:10.1080/0891693031000152688 (2003).

118 Zhang, S. C. & Fedoroff, S. Cellular localization of stem cell factor and c-kit receptor in the mouse nervous system. *Journal of Neuroscience Research* **47**, 1-15 (1997).

119 Zhang, S. C. & Fedoroff, S. Expression of stem cell factor and c-kit receptor in neural cells after brain injury. *Acta Neuropathol.* **97**, 393-398 (1999).

120 Chen, R. H. *et al.* A Hierarchy of Self-Renewing Tumor-Initiating Cell Types in Glioblastoma. *Cancer Cell* **17**, 362-375, doi:10.1016/j.ccr.2009.12.049 (2010).

121 Wei, J. *et al.* Glioma-Associated Cancer-Initiating Cells Induce Immunosuppression. *Clin. Cancer Res.* **16**, 461-473, doi:10.1158/1078-0432.ccr-09-1983 (2010).

122 Hussain, S. F. *et al.* The role of human glioma-infiltrating microglia/macrophages in mediating antitumor immune responses. *Neuro-Oncology* **8**, 261-279, doi:10.1215/15228517-2006-008 (2006).

123 Saura, J. Microglial cells in astroglial cultures: a cautionary note. *Journal of Neuroinflammation* **4**, doi:10.1186/1742-2094-4-26 (2007).

124 Ignacio, A. R., Muller, Y. M. R., Carvalho, M. S. L. & Nazari, E. M. Distribution of microglial cells in the cerebral hemispheres of embryonic and neonatal chicks. *Brazilian Journal of Medical and Biological Research* **38**, 1615-1621 (2005).

125 Lyck, L., Bulavina, L., and Finsen, B. New parameters for analysis of changes microglial morphology using stereology and histomorphometry. *FASEB J.* **23** (2009).

126 Azevedo, F. A. C. *et al.* Equal Numbers of Neuronal and Nonneuronal Cells Make the Human Brain an Isometrically Scaled-Up Primate Brain. *Journal of Comparative Neurology* **513**, 532-541, doi:10.1002/cne.21974 (2009).

127 Pelvig, D. P., Pakkenberg, H., Stark, A. K. & Pakkenberg, B. Neocortical glial cell numbers in human brains. *Neurobiology of Aging* **29**, 1754-1762, doi:10.1016/j.neurobiolaging.2007.04.013 (2008).
128 Allen, N. J. & Barres, B. A. NEUROSCIENCE Glia - more than just brain glue. *Nature* **457**, 675-677, doi:10.1038/457675a (2009).
129 Vergara, D. *et al.* Biomechanical and proteomic analysis of INF- beta-treated astrocytes. *Nanotechnology* **20**, 455106 (2009).
130 Hatten, M. E. NEURONAL REGULATION OF ASTROGLIAL MORPHOLOGY AND PROLIFERATION INVITRO. *Journal of Cell Biology* **100**, 384-396 (1985).
131 Wilson, A. *et al.* Hematopoietic Stem Cells Reversibly Switch from Dormancy to Self-Renewal during Homeostasis and Repair. *Cell* **135**, 1118-1129, doi:10.1016/j.cell.2008.10.048 (2008).
132 Jellinger, K. GLIOBLASTOMA MULTIFORME - MORPHOLOGY AND BIOLOGY. *Acta Neurochirurgica* **42**, 5-32 (1978).
133 Meeker, R. B., Azuma, Y., Bragg, D. C., English, R. V. & Tompkins, M. Microglial proliferation in cortical neural cultures exposed to feline immunodeficiency virus. *Journal of Neuroimmunology* **101**, 15-26 (1999).
134 Shankaran, M. *et al.* Measurement of brain microglial proliferation rates in vivo in response to neuroinflammatory stimuli: Application to drug discovery. *Journal of Neuroscience Research* **85**, 2374-2384, doi:10.1002/jnr.21389 (2007).
135 Koguchi, K., Nakatsuji, Y., Okuno, T., Sawada, M. & Sakoda, S. Microglial cell cycle-associated proteins control microglial proliferation in vivo and in vitro and are regulated by GM-CSF and density-dependent inhibition. *Journal of Neuroscience Research* **74**, 898-905, doi:10.1002/jnr.10829 (2003).
136 Vergara, D. *et al.* Biomechanical and proteomic analysis of INF-beta-treated astrocytes. *Nanotechnology* **20**, doi:10.1088/0957-4484/20/45/455106 (2009).
137 Stoker, M. G. P. & Rubin, H. DENSITY DEPENDENT INHIBITION OF CELL GROWTH IN CULTURE. *Nature* **215**, 171-& (1967).
138 Laterra, J. *et al.* Scatter factor hepatocyte growth factor gene transfer enhances glioma growth and angiogenesis in vivo. *Laboratory Investigation* **76**, 565-577 (1997).
139 Paugh, B. S. *et al.* Interleukin-1 Regulates the Expression of Sphingosine Kinase 1 in Glioblastoma Cells. *Journal of Biological Chemistry* **284**, 3408-3417, doi:10.1074/jbc.M807170200 (2009).
140 Tada, M. & Detribolet, N. RECENT ADVANCES IN IMMUNOBIOLOGY OF BRAIN-TUMORS. *Journal of Neuro-Oncology* **17**, 261-271 (1993).
141 Jennings, M. T. *et al.* TGF-BETA-1 AND TGF-BETA-2 ARE POTENTIAL GROWTH-REGULATORS FOR LOW-GRADE AND MALIGNANT GLIOMAS INVITRO - EVIDENCE IN SUPPORT OF AN AUTOCRINE HYPOTHESIS. *International Journal of Cancer* **49**, 129-139 (1991).
142 Jennings, M. T. & Pietenpol, J. A. The role of transforming growth factor beta in glioma progression. *Journal of Neuro-Oncology* **36**, 123-140 (1998).
143 Platten, M., Wick, W. & Weller, M. Malignant glioma biology: Role for TGF-beta in growth, motility, angiogenesis, and immune escape. *Microscopy Research and Technique* **52**, 401-410 (2001).
144 Keller, G. & Snodgrass, R. LIFE-SPAN OF MULTIPOTENTIAL HEMATOPOIETIC STEM-CELLS INVIVO. *Journal of Experimental Medicine* **171**, 1407-1418 (1990).
145 Seruya, M. *et al.* Clonal population of adult stem cells: Life span and differentiation potential. *Cell Transplantation* **13**, 93-101 (2004).
146 Simonsen, J. L. *et al.* Telomerase expression extends the proliferative life-span and maintains the osteogenic potential of human bone marrow stromal cells. *Nature Biotechnology* **20**, 592-596, doi:10.1038/nbt0602-592 (2002).

147 Bodnar, A. G. *et al.* Extension of life-span by introduction of telomerase into normal human cells. *Science* **279**, 349-352 (1998).
148 Ross, H. J., Sato, N., Ueyama, Y. & Koeffler, H. P. CYTOKINE MESSENGER-RNA STABILITY IS ENHANCED IN TUMOR-CELLS. *Blood* **77**, 1787-1795 (1991).
149 Beutler, B. A., Milsark, I. W. & Cerami, A. CACHECTIN TUMOR NECROSIS FACTOR - PRODUCTION, DISTRIBUTION, AND METABOLIC-FATE INVIVO. *Journal of Immunology* **135**, 3972-3977 (1985).
150 Blick, M., Sherwin, S. A., Rosenblum, M. & Gutterman, J. PHASE-I STUDY OF RECOMBINANT TUMOR-NECROSIS-FACTOR IN CANCER-PATIENTS. *Cancer Research* **47**, 2986-2989 (1987).
151 Chapman, P. B. *et al.* CLINICAL-PHARMACOLOGY OF RECOMBINANT HUMAN-TUMOR NECROSIS FACTOR IN PATIENTS WITH ADVANCED CANCER. *Journal of Clinical Oncology* **5**, 1942-1951 (1987).
152 Fong, Y., Moldawer, L. L., Shires, G. T. & Lowry, S. F. THE BIOLOGIC CHARACTERISTICS OF CYTOKINES AND THEIR IMPLICATION IN SURGICAL INJURY. *Surgery Gynecology & Obstetrics* **170**, 363-378 (1990).
153 Schetz, M., Ferdinande, P., Vandenberghe, G., Verwaest, C. & Lauwers, P. REMOVAL OF PRO-INFLAMMATORY CYTOKINES WITH RENAL REPLACEMENT THERAPY - SENSE OR NONSENSE. *Intensive Care Medicine* **21**, 169-176 (1995).
154 Glauche, I. *et al.* Stem Cell Proliferation and Quiescence-Two Sides of the Same Coin. *Plos Computational Biology* **5**, doi:e1000447

10.1371/journal.pcbi.1000447 (2009).
155 Horsley, V., Aliprantis, A. O., Polak, L., Glimcher, L. H. & Fuchs, E. NFATc1 balances quiescence and proliferation of skin stem cells. *Cell* **132**, 299-310, doi:10.1016/j.cell.2007.11.047 (2008).
156 Venezia, T. A. *et al.* Molecular signatures of proliferation and quiescence in hematopoietic stem cells. *Plos Biology* **2**, 1640-1651, doi:e301

10.1371/journal.pbio.0020301 (2004).
157 Higuchi, M., Ohnishi, T., Arita, N., Hiraga, S. & Hayakawa, T. EXPRESSION OF TENASCIN IN HUMAN GLIOMAS - ITS RELATION TO HISTOLOGICAL MALIGNANCY, TUMOR DEDIFFERENTIATION AND ANGIOGENESIS. *Acta Neuropathol.* **85**, 481-487 (1993).
158 Dai, C. *et al.* PDGF autocrine stimulation dedifferentiates cultured astrocytes and induces oligodendrogliomas and oligoastrocytomas from neural progenitors and astrocytes in vivo. *Genes Dev.* **15**, 1913-1925, doi:10.1101/gad.903001 (2001).
159 van Veelen, M. L. C., Avezaat, C. J. J., Kros, J. M., van Putten, W. & Vecht, C. Supratentorial low grade astrocytoma: prognostic factors, dedifferentiation, and the issue of early versus late surgery. *J. Neurol. Neurosurg. Psychiatry* **64**, 581-587, doi:10.1136/jnnp.64.5.581 (1998).
160 Niwa, H., Miyazaki, J. & Smith, A. G. Quantitative expression of Oct-3/4 defines differentiation, dedifferentiation or self-renewal of ES cells. *Nature Genet.* **24**, 372-376, doi:10.1038/74199 (2000).
161 Zhang, Q. B. *et al.* Differentiation profile of brain tumor stem cells: a comparative study with neural stem cells. *Cell Res.* **16**, 909-915, doi:10.1038/sj.cr.7310104 (2006).
162 Araten, D. J. *et al.* A quantitative measurement of the human somatic mutation rate. *Cancer Research* **65**, 8111-8117, doi:10.1158/0008-5472.can-04-1198 (2005).
163 Tomlinson, I. P. M., Novelli, M. R. & Bodmer, W. F. The mutation rate and cancer. *Proceedings of the National Academy of Sciences of the United States of America* **93**, 14800-14803, doi:10.1073/pnas.93.25.14800 (1996).
164 Jackson, A. L. & Loeb, L. A. The mutation rate and cancer. *Genetics* **148**, 1483-1490 (1998).

165 Benveniste, E. N., Tang, L. P. & Law, R. M. DIFFERENTIAL REGULATION OF ASTROCYTE TNF-ALPHA EXPRESSION BY THE CYTOKINES TGF-BETA, IL-6 AND IL-10. *International Journal of Developmental Neuroscience* **13**, 341-349 (1995).

166 Takano, S. *et al.* Concentration of vascular endothelial growth factor in the serum and tumor tissue of brain tumor patients. *Cancer Research* **56**, 2185-2190 (1996).
